# Strategies to Stabilize the Photoinduced Supramolecular Chirality in Azobenzene Liquid Crystalline Polymers

**DOI:** 10.3390/polym11050885

**Published:** 2019-05-15

**Authors:** Jorge Royes, Luis Oriol, Rosa M. Tejedor, Milagros Piñol

**Affiliations:** 1 Departamento de Química Orgánica, Facultad de Ciencias, Instituto de Ciencia de Materiales de Aragón (ICMA), Universidad de Zaragoza-CSIC, c/ Pedro Cerbuna 12, 50009 Zaragoza, Spain; jorge.royes@ens.fr (J.R.); loriol@unizar.es (L.O.); 2 UMR 8640, CNRS, École Normale Superieure Département de Chimie, 24 rue Lhomond, 75005 Paris, France; 3 Centro Universitario de la Defensa, Academia General Militar, Ctra. de Huesca s/n, 50090 Zaragoza, Spain

**Keywords:** liquid crystallinity, light induced chirality, photoactive polymers, azobenzene, cinnamate ester

## Abstract

This paper describes the synthesis, thermal characterization and optical properties of liquid crystalline homopolymers and block copolymers with a repeating unit consisting of two functional units, with at least one of them being an azobenzene. Films of these polymers have been irradiated with circularly polarized light at room temperature, evaluating the intensity of the photoinduced chiral signal and its temporal stability upon storage. The paper also explores two different strategies to restrict the relaxation of the photoinduced order. Firstly, block copolymers have been prepared to confine the photoaddressable segments into nanoscopic domains where relaxation should be restricted. Secondly, an alternative homopolymer has been synthesized where the repeating unit combines two chromophores that can be separately photoaddressed, an azobenzene unit to efficiently photoinduce chirality and a cinnamate to fix the chiral signal by photocrosslinking.

## 1. Introduction

Azobenzene is known to photoisomerize in a reversible way with ultraviolet-visible (UV–Vis) light from the thermodynamically stable *E*-isomer to the *Z*-isomer. The repeated photoinduction of *E*-*Z*-*E* isomerization cycles with suitable wavelength, intensity or polarization has been used to generate order and anisotropy at different levels in azobenzene films that eventually culminate in phenomena such as photoinduced birefringence, photoinduced chirality or surface relief grating formation, which are the basis for different optical devices [1,2,3,4].

Amongst different architectures, poly(meth)acrylates with pendant azobenzene moieties have been profusely investigated for different optical applications, with the magnitude and stability of the photoinduced response being strongly dependent not only on the electronic nature of the azobenzene substituents but also on the way in which azobenzenes are bound to the polymeric chain [5]. For instance, azobenzene units are frequently attached to the poly(meth)acrylate chain by flexible alkyl spacers, decoupling their movements from those of the polymer chain and, therefore, facilitating an efficient photorientation. Besides, imparting liquid crystalline properties is a common strategy to enhance the properties of photoresponsive azopolymers because the intrinsic self-organization ability of liquid crystalline polymers usually increases the photoinduced order and its temporal stability compared to amorphous ones [6]. Even though substituted *E*-azobenzenes might behave both as mesogenic and photoresponsive moieties, copolymerization of azobenzene and photopassive mesogenic monomers (commonly biphenyls or tolanes) has been reported to amplify the photoinduced effects. In statistical copolymers, light induced ordering of azobenzenes is transferred to photopassive mesogenic groups through cooperative molecular interplays operating at the liquid crystalline state [7,8]. The additional advantage of copolymers having units that do not absorb at the azobenzene wavelength is the possibility of processing thicker films required for optical applications such as volume holography.

Copolymerization opens the door not only to the inclusion of optically inert monomers but also to the inclusion of several photoactive moieties. As azobenzenes, cinnamates have also been incorporated into side chain liquid crystalline polymers to control the molecular orientation and photoinduce linear birefringence [4,8,9]. Cinnamate esters can undergo different photochemical processes such as *E-Z* photoisomerization, photo-Fries rearrangement and [2+2] photocycloaddition but they suffer from limited reversibility efficiency [9,10,11,12]. Merging both chromophores into the same liquid crystalline copolymer has already been explored to create materials with linear anisotropy where the azobenzene efficiently captures the anisotropy of polarized light while the cinnamate ester permanently fixes the photoinduced order by [2+2] photocycloaddition [13,14].

Diblock copolymers with two dissimilar polymeric segments linked by a covalent bond can lead to microphase segregation yielding different nanostructures. Photoresponsive liquid crystalline block copolymers integrating the light-addressable features of azobenzene, self-organization of liquid crystals and microphase separation of well-defined block copolymers have emerged as a strategy towards the generation of hierarchically ordered structures with optical activity [15,16,17]. When the liquid crystalline photoresponsive block forms the minority phase, the photoinduced response can be confined into nanoscopic domains dispersed into an inert continuous matrix using for example poly(ethylene oxide) or poly(methyl methacrylate) as the majority phase [18]. Thus, confinement preserves proximity and cooperative molecular motions of the photoaddressable moieties, as in homopolymers, ensuring an optimal photoresponse. In addition to their lowered absorption at working wavelength, if the nanoscopic domains are smaller than 100 nm, scattering of visible light can also be drastically reduced, improving the optical performance of the material. The confinement also has an effect on the photoinduced orientation, as changes in organization involving motion and rearrangement of azobenzene moieties are more difficult to develop inside microdomains [19].

It has been proven for some time that by using circularly polarized light (CPL), a chiral supramolecular organization of the azobenzene chromophores can be induced in achiral azopolymers with a sense that is governed by the handedness of the incident beam [20]. The interaction with CPL depends on structural differences as well as the amorphous/liquid crystalline nature of the polymeric materials and the glass transition temperature (T_g_) [21] Because the generation of chiral organizations in bulk requires of enough molecular mobility of the azobenzene units, for intrinsically chiral polymers, the amplification of the chiral organization has been achieved by thermal annealing close to T_g_ [22]. However, when the chiral supramolecular organization is promoted by light illumination, the mobility is probably favored by a decrease on the T_g_ of the polymer when the *E*-azobenzene is converted into the *Z*-azobenzene [23]. We have studied this phenomenon on a series of azobenzene polymethacrylate homopolymers and related block copolymers. The polymers were prepared by combining the atom transfer radical polymerization (ATRP) of poly(propargyl methacrylate) with postfunctionalization by copper(I) catalyzed alkyne-azide cycloaddition (CuAAC) using azobenzene azides [24,25,26,27]. With this advantageous combination, it was possible to efficiently change the nature of the functional moieties. The key feature of the investigated polymers was the presence of two functional units in the repeating unit, with at least one of them being a 4-cyanoazobenzene. The modulation of the thermal and optical properties was attained just by varying the nature of the second functional unit. According to the results, for liquid crystalline homopolymers the magnitude of the chiral signal and the extension of the photoinduced chiral order significantly depended on the T_g_ values. If the T_g_ was close to room temperature (T_g_ ≈ 28–18 °C), which is also the irradiation temperature, the photoinduced chiral response was lost in a few hours upon storage at room temperature [24]. When the T_g_ was well above room temperature (T_g_ ≈ 50–70 °C) [27] the chiral response was approximately of the same magnitude as if the liquid crystalline properties were preserved, but the photoinduced chiral order was maintained for months. However, for amorphous polymers that lack liquid crystalline behavior, the photoinduced chiral signal was about one order of magnitude less intense despite having a similar temporal stability. Besides, no chiral order was photoinduced in related block copolymers having very low volume fractions of the azobenzene segment and with no liquid crystalline properties [27].

In this work, we explore the photoinduction and retention of chirality on liquid crystalline homopolymers with T_g_ values just above room temperature (T_g_ ≈ 30–50 °C). Furthermore, the confinement of the liquid crystalline segments into microdomains, as in block copolymers, and crosslinking after the chiral photoinduction are explored as strategies to stabilize the chiral order.

## 2. Materials and Methods

### 2.1. General Synthetic Procedures

Details of the synthesis and characterization of the azides are given in the supplementary information. The preparation of poly(propagyl methacrylate)s **P_70_** and **P_100_** and their characterization have been described elsewhere [25,26]. Postpolymerization functionalization of the polymers with the azides by CuAAC was accomplished using previously reported conditions [24]. Methyl methacrylate from Sigma-Aldrich (Sigma-Aldrich Gmbh, Steinheim, Germany) was filtered through a pad of basic alumina, dried over CaH_2_ and distilled prior to use. 3-(Trimethylsilyl)propargyl methacrylate was prepared according to literature procedures [28]. 1,1,4,7,7-Pentamethyldiethylenetriamine (PMDETA), ethyl 2-bromoisobutyrate (EBB) and copper(I) bromide from Sigma-Aldrich were used as received. Toluene and *N*,*N*-dimethylformamide (DMF) were dried over CaH_2_ for 72 h, distilled and deoxygenated by bubbling argon through for 20 min. ^1^H NMR spectra of polymers are collected at the supporting information (Figures S1–S5).

#### 2.1.1. Characterization Data of Functionalized Homopolymers

**P_100_(7eAZO/eAZO)**. IR (KBr disk, cm^−1^): 2228 (C≡N), 1744 (C=O). ^1^H NMR (400 MHz, CDCl_3_, δ, ppm): 8.15–7.76, 7.73–7.61, 6.99–6.80, 5.37–4.95, 4.85–4.66, 4.64–4.43, 4.34–4.17, 4.17–4.04, 3.99–3.82, 2.37–2.15, 1.86–1.64, 1.59–1.48, 1.46–1.10, 0.93–0.50. Elem. Anal. Exp. (calc. for C_56_H_63_N_9_O_10_): C 65.50% (65.80%), H 6.51% (6.21%), N 12.08% (12.33%). SEC (5 × 10^−2^ M LiBr in DMF): *M_n_*^SEC^ = 84 × 10^3^ g mol^−1^, *Ð* = 1.19.

**P_100_(10cCNB/eAZO)**. IR (KBr disk, cm^−1^): 3404 (N–H), 2226 (C≡N), 1745 (C=O). ^1^H NMR (400 MHz, CDCl_3_, δ, ppm): 8.15–7.96, 7.92–7.79, 7.78–7.65, 7.65–7.49, 7.48– 7.37, 7.02–6.82, 5.47–5.33, 5.22–4.97, 4.88–4.39, 4.31–4.03, 4.02–3.81, 3.19–2.88, 2.34–2.15, 1.87–1.04, 0.93–0.45. Elem. Anal. Exp. (calc. for C_61_H_74_N_8_O_10_): C 67.32% (67.88%), H 7.17% (6.91%), N 10.52% (10.38%). SEC (5 × 10^−2^ M LiBr in DMF): *M_n_^SEC^* = 91 × 10^3^ g mol^−1^, *Ð* = 1.18.

**P_70_(7cCIN/cAZO)**. IR (KBr disk, cm^−1^): 3337 (N–H), 2225 (C≡N), 1736 (C=O). ^1^H NMR (400 MHz, CDCl_3_, δ, ppm): 8.08–7.69, 7.68–7.53, 7.52–7.42, 7.03–6.83, 5.40–4.91, 4.81–4.62, 4.65–4.55, 4.26–4.06, 4.06–3.84, 3.22–2.92, 2.00–1.67, 1.67–1.53, 1.51–1.07, 1.05–0.45. Elem. Anal. Exp. (calc. for C_60_H_73_N_9_O_10_): C 67.44% (67.71%), H 6.88% (6.81%), N 11.12% (11.67%). SEC (5 × 10^−2^ M LiBr in DMF): *M_n_^SEC^* = 73 × 10^3^ g mol^−1^, *Ð* = 1.19.

#### 2.1.2. Synthesis and Characterization of Functionalized Block Copolymers

Synthesis of **P_40_TMS**. A solution of 3-(trimethylsilyl)propargyl methacrylate (8.341 g, 42.3 mmol), PMDETA (77.2 μL, 0.4 mmol) and EBB (53.4 μL, 0.4 mmol) in freshly distilled toluene (20 mL) was added via cannula to a Schlenk flask charged with CuBr (53.0 mg, 0.4 mmol) under argon atmosphere. Oxygen was removed by successive freeze-pump-thaw cycles by applying vacuum and backfilling with argon. Polymerization was maintained at 70 °C for 24 h, then the mixture was diluted with THF and filtered through a pad of neutral alumina. The solution was stirred with DOWEX Marathon® acid exchange resin for 4 h at room temperature. Then, the resin was filtered off and the volatiles were removed in a rotary evaporator. The residue was taken into the minimum amount of THF and precipitated in a cold methanol/water (10:2) mixture. The polymer was isolated as a white powder by filtration and dried under vacuum (1.584 g, 19% yield). IR (KBr disk, cm^−1^): 2189 (C≡C), 1742 (C=O), 852, 762 (Si–C). ^1^H NMR (400 MHz, CDCl_3_, δ, ppm): 4.73–4.58, 2.19–1.69, 1.65–0.76, 0.34–0.08. SEC (THF): *M_n_^SEC^* = 8.5 × 10^3^ g mol^−1^, *Ð* = 1.27 Polymerization degree, n = 41 ± 3, was calculated by ^1^H NMR analysis of terminal groups performed in 3 independent samples of polymer using the relative integration of peaks centered at 4.09 ppm (corresponding to the terminal EBB initiator residue) and at 4.60 ppm (corresponding to –COOC*H*_2_C≡C– of the repeating unit). *M_n_^NMR^* ≈ 8.0 ± 0.6 × 10^3^ g mol^−1^.

Synthesis of **P_40_TMS-*b*-PMMA_800_**. A solution of methyl methacrylate (4.235 g, 42.3 mmol), **P_40_TMS** (53.4 mg, 0.4 mmol) and PMDETA (77.2 μL, 0.4 mmol) in toluene (20 mL) was transferred to a Schlenk flask with CuBr (53.0 mg, 0.4 mmol) under argon atmosphere. After oxygen removal by successive freeze-pump-thaw cycles, the polymerization was maintained for 24 h at 70 °C. The reaction mixture was diluted with THF filtered through neutral alumina and stirred with DOWEX Marathon® acid exchange resin for 4 h at room temperature. Then, the resin was filtered off and the volatiles removed in a rotary evaporator. The residue was taken into the minimum amount of THF and precipitated in a cold methanol/water (10:2) mixture. The polymer was isolated as a white powder by filtration and dried under vacuum (1.638 g, 21% yield). IR (KBr disk, cm^−1^): 2199 (C≡C), 1735 (C=O), 847, 754 (Si–C). 1H NMR (400 MHz, CDCl3, δ, ppm): 4.69–4.50, 3.72–3.45, 2.14–1.69, 1.51–0.70, 0.28–0.09. *M_n_^SEC^* = 109 × 10^3^ g mol^−1^, *Ð* = 1.30.

Synthesis of **P_40_-*b*-PMMA_800_**. A solution of **P_40_TMS-*b*-PMMA_800_** and glacial acetic acid (1.5 mol per mole of alkyne) in dry THF (≈15 mL per mg of polymer) was cooled in an ice-salt bath. Then, 0.2 M tetrabutylammonium fluoride (TBAF) in THF was dropwise added (1.5 moles per alkyne mole). The reaction was warmed up to room temperature while the extent of the reaction was followed by ^1^H NMR. After 24 h, the mixture was filtered through a silica gel pad and the solvent eliminated in a rotary evaporator. The residue was dissolved in the minimum amount of THF and precipitated into methanol. The polymer was isolated by filtration and dried under vacuum (85% yield). IR (KBr disk, cm^−1^): 3300 (≡C–H), 2133 (C≡C), 1736 (C=O). ^1^H NMR (400 MHz, CDCl_3_, δ, ppm): 4.71–4.53, 3.72–3.45, 2.58–2.42, 2.24–1.61, 1.52–0.69. *M_n_^SEC^* = 95 × 10^3^ g mol^−1^, *Ð* = 1.27. The polymerization degree of the PMMA segment, m = 773 ± 8, was calculated by ^1^H NMR using the relative integration of peaks centered at 2.50 ppm (corresponding to –C≡CH of the propargyl methacrylate repeating unit) and at 3.60 ppm (corresponding to –COOC*H*_3_ of the methy methacrylate repeating unit) taking into account the polymerization degree (n = 41) previously determined for the **P_40_TMS** block, given a *M_n_^NMR^* = 79.7 ± 0.8 × 10^3^ g mol^−1^.

Synthesis of functional block copolymers. General procedure: A solution of PMDETA (10.4 μL, 0.05 mmol) in distilled and deoxygenated DMF (2 mL) was added to a Schlenk flask containing the azide, either **N_3_–7eAZO/eAZO** or **N_3_–10cCNB/eAZO** (0.1 mmol), **P_40_-*b*-PMMA_700_** (121.3 mg, 0.05 mmol of alkyne groups) and CuBr (7.2 mg 0.05 mmol). Oxygen was removed by successive freeze-pump-thaw cycles and the reaction was maintained at 35 °C for 3 days. Then, it was diluted with THF, filtered through a pad of neutral alumina and stirred with DOWEX Marathon^®^ acid exchange resin for 4 h at room temperature. After removal of the resin, the solvent was evaporated, and the crude purified by preparative size exclusion chromatography (SEC) on Bio-Beads^®^ S-X1 and THF as the eluent. The residue was dissolved into the minimum amount of THF and precipitated into methanol. **P_40_(7eAZO/eAZO)-*b*-PMMA_800_** was obtained as an orange powder (83% yield). IR (KBr disk, cm^−1^): 2230 (C≡N), 1740 (C=O). ^1^H NMR (400 MHz, CDCl_3_, δ, ppm): 8.12–7.90, 7.87–7.71, 7.71–7.55, 7.03–6.64, 5.16 –4.84, 4.76–4.39, 4.24–4.01, 3.98–3.79, 3.62–3.40, 2.30–2.09, 2.01–1.58, 1.56–0.55. Elem. Anal. Exp. (calc. for C_171_H_247_N_9_O_56_): C 61.86% (61.77%), H 7.49% (7.49%), N 3.59% (3.79%). SEC (THF): *M_n_^SEC^* = 150 × 10^3^ g mol^−1^, *Ð* = 1.30. Average number molar mass based on the average polymerization degrees determined for **P_40_TMS-*b*-PMMA_800_** by ^1^H NMR, n = 41 and m = 773, assuming that all alkyne groups have reacted, *M_n_^NMR^* ≈ 114 × 10^3^ g mol^−1^. **P_40_(10cCNB/eAZO)-*b*-PMMA_800_** was obtained as an orange powder (76% yield). IR (KBr disk, cm^−1^): 3347 (N–H), 2228 (C≡N), 1736 (C=O). ^1^H NMR (400 MHz, CDCl_3_, δ, ppm): 8.10–7.80, 7.78–7.65, 7.66–7.51, 7.49–7.36, 7.04–6.81, 5.50–5.25, 5.21–4.90, 4.81–4.41, 4.29–4.04, 4.02–3.86, 3.80–3.40, 3.18–2.93, 2.36–2.15, 2.11–1.66, 1.59–0.59. Elem. Anal. Exp. (calc. for C_176_H_258_N_8_O_56_): C 62.36% (62.51%), H 7.86% (7.69%), N 3.53% (3.33%). SEC (THF): *M_n_^SEC^* = 120 × 10^3^ g mol^−1^, *Ð* = 1.30. Average number molar mass based on the average polymerization degrees determined for **P_30_TMS-b-PMMA_800_** by ^1^H NMR, n = 41 and m = 773, assuming that all alkyne groups have reacted, *M_n_^NMR^* ≈ 121 × 10^3^ g mol^−1^.

### 2.2. Film Processing and Irradiation

Polymer films were prepared by casting a chloroform solution (1.5 mg mL^−1^) of the polymer onto clean, fused silica slides to have maximum absorbance values between 1.0 and 2.0 in the 220–400 nm interval. The as-casted films were dried under vacuum at 30 °C for 24 h, then thermally annealed and rapidly cooled down to room temperature. Thermal annealing conditions depended on the thermal properties of the polymer. Liquid crystalline homopolymers were first annealed for 30 min at the isotropic state (T = 1.2 × T_i_, where T_i_ is the isotropization temperature) then 30 min at the liquid crystalline state (T = 0.7 × T_i_), and rapidly quenched to room temperature to vitrify the mesophase. Block copolymers were subjected to the same temperature profiles but prolonging treatments at the mesophase or above T_g_ for 2 h. Films were stored in the dark at 30 °C.

Annealed films were irradiated with circularly polarized light (CPL) for 30 min using the 488 nm line of an Ar^+^ Stellar Pro Laser from Modu-Laser (Modu-Laser, Centerville, UT, USA) coupled to a beam expansor-collimator and a quarter-wave plate (20 mW cm^−2^) (Figure S10) [20]. A circular mask of 5 mm diameter was used to limit the irradiated area. Quality of CPL was assessed measuring the variation of light intensity in function of the rotation of a linear polarizer (relation between minimum and maximum intensity = 0.96). All the films were firstly irradiated with right handed CPL (r-CPL), thermally annealed to erase the photoinduced chiral order, and then irradiated with left handed CPL (l-CPL). Electronic circular dichroism (ECD) and UV-vis spectra of the films were registered before and after irradiation using a Jasco J 810 spectropolarimeter (Jasco, Tokyo, Japan). ECD spectra were registered by rotating the film every 60° around the light beam axis in order to check that the contribution of linear dichroism to ECD spectra was negligible. Film of **P_70_(7cCIN/eAZO)** was irradiated with UV light using a mercury (Hg) UV-VIS lamp Oriel (1000 W) with water filter and a bandpass filter with a transmittance range 260–350 nm (47 mW cm^−2^).

### 2.3. Techniques

Proton Nuclear Magnetic Resonance (^1^H NMR) spectra were acquired on a 400 MHz Bruker AV-400 spectrometer (Bruker, Billerica, MA, USA) using CDCl_3_. In order to get quantitative information, the NMR spectra of the polymers were acquired in a static non-spinning mode, using a 90° excitation pulse with a long T1 relaxation time (10 s + 5 s acquisition). Fourier Transform Infrared (FT-IR) spectra were collected using a Bruker Tensor 27 (Bruker, Billerica, MA, USA) in KBr disks. Elemental analyses were performed using a Perkin-Elmer 240C microanalyzer (PerkinElmer, Waltham, MA, USA). Size Exclusion Chromatography (SEC) was performed using a Waters 2695 liquid chromatography system equipped with a Waters 2420 Evaporative Light Scattering detector (Waters, Milford, MA, USA) and/or a UV-vis-Waters 2998 Photodiode Array detector. SEC analysis of homopolymers **P_100_(7eAZO/eAZO)**, **P_100_(10cCNB/eAZO)** and **P_70_(7cCIN/cAZO)** were carried out in DMF (containing 5 × 10^−2^ M LiBr) at 65 °C and 0.5 mL min^−1^ flow rate, using a combination of two Styragel® columns (HR1 and HR3 from Waters) providing an effective molar mass range of 100–6 × 10^5^ g mol^−1^ and applying a calibration with polystyrene standards. SEC analysis of block copolymers **P_40_(7eAZO/eAZO)-*b*-PMMA_800_** and **P_40_(10cCNB/eAZO)-*b*-PMMA_800_** were carried out in THF at 35 °C and 1 mL min^−1^ flow rate, combining two PLgel columns (5 mm Mixed C and 5 mm Mixed E from Agilent Technologies) providing an effective molar mass range of 100–2 × 10^6^ g mol^−1^ and applying a calibration with poly(methyl methacrylate) standards.

Differential scanning calorimetry (DSC) was performed using a DSC Q2000 from TA Instruments (TA Instruments, New Castle, DE, USA) with samples (approx. 3 mg) sealed in aluminium pans at a scanning rate of 10 or 20 °C min^−1^. Temperatures were read at the maximum of the transition peaks, and the glass transition temperature (T_g_) was read at the midpoint of the heat capacity increase. Thermogravimetric analysis (TGA) was performed at 10 °C min^−1^ under nitrogen atmosphere using an SDT 2960 Simultaneous DTA-TGA from TA Instruments. TGA data were given as the onset of the decomposition curve, and DTGA data were given at the maxima of the weight first derivative curve peaks.

The optical textures of the mesophases were studied with an Olympus BH-2 polarizing microscope equipped with a Linkam THMS600 (Linkam Scientific, Tadworth, UK) hot stage and a TMS91 cooling system (Linkam Scientific, Tadworth, UK). X-ray diffraction (XRD) measurements were performed with an evacuated Pinhole camera (Anton-Paar) operating (Anton Paar, Graz, Austria) with a point-focused Ni-filtered Cu-Kα beam. Powdered samples were placed in quartz Lindemann capillaries of 1 mm diameter. The patterns were collected on flat photographic films perpendicular to the X-ray beam.

Images by transition electron microscopy (TEM) were obtained in a Tecnai T20 operating (FEI ThermoFisher Scientific, Waltham, MA, USA) at 200 kV. Samples were prepared by heating the powdery polymer (2–3 mg) at 180 °C for 5 min to get a small compact pellet of the molten polymer that was annealed for 24 h at 150 °C under vacuum. The annealed pellet was embedded in the epoxy resin EMbed 812 and then ultramicrotomed to get ultrathin slides of approx. 100 nm thickness that were picked up on carbon-coated copper grids. The slices were exposed to RuO_4_ vapors for 45 min.

## 3. Results and Discussion

### 3.1. Synthesis and Thermal Characterization of Azides

**Figure 1:**
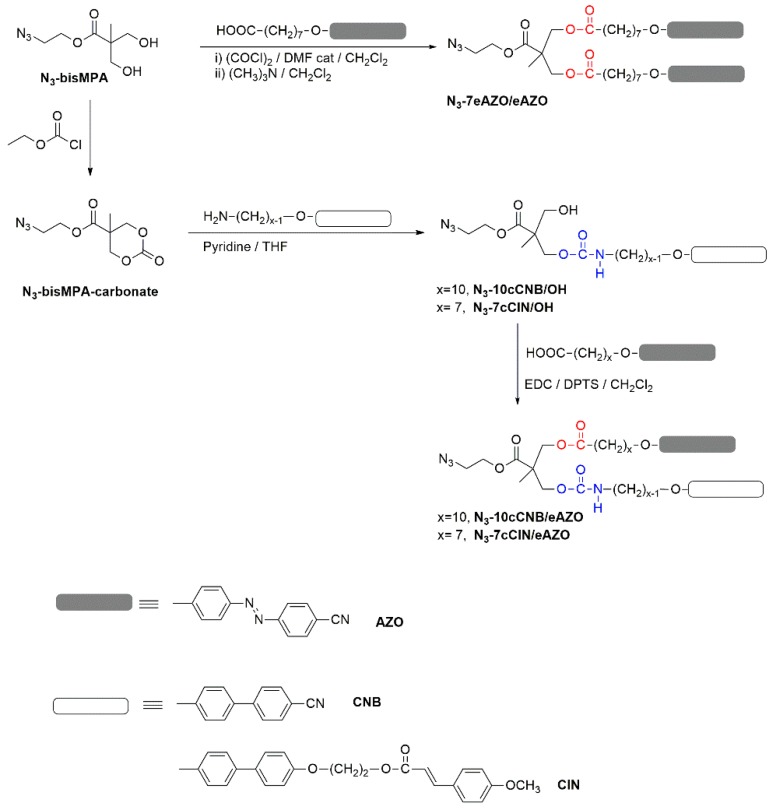
General road map for the synthesis of azides series.

Azides with two functional units were prepared using 2,2-bis(hydroxymethyl)propionic acid (bisMPA) as the central linker employing the carboxylic group to introduce the clickable azide group and the two hydroxyl groups to introduce 4-cyanoazobenzene (AZO), 4-methoxycinnamoyl (CIN) or 4-cyanobiphenyl (CNB) units through ester or carbamate binding groups. Figure 1 summarizes the general road map that gives access to them. Full experimental details and characterization data are collected in the supporting information. Azide **N_3_–7eAZO/eAZO** with two azobenzene units, where ‘7’ stands for the length of the flexible spacer and ‘e’ for the ester linking group, was easily prepared by esterification of the bisMPA hydroxyl groups with the corresponding acyl chloride in a 1:2 molar ratio. The synthesis of the azides with two different functional moieties, either CNB/AZO or CIN/AZO, was approached as described by Fréchet et al. [29] using an intermediate cyclic carbonate that was opened with a primary amine forming a carbamate binding group, giving rise to **N_3_–10cCNB/OH** or **N_3_–7cCIN/OH** intermediate azides where ‘c’ stands for the carbamate linking group. The remainder hydroxyl group was subsequently esterified under Steglich conditions, rendering target azides **N_3_–10cCNB/eAZO** and **N_3_-7cCIN/eAZO**.

The liquid crystalline properties of the azides were studied by differential scanning calorimetry (DSC) and polarized optical microscopy (POM), and results are summarized in Table 1. Azide **N_3_–7eAZO/eAZO** showed both nematic and smectic A mesophases while **N_3_–10cCNB/eAZO** and **N_3_–7cCIN/eAZO** formed a stable smectic A phase that vitrified on cooling forming mesomorphic glasses.

### 3.2. Synthesis and Characterization of Polymers

**Figure 2:**
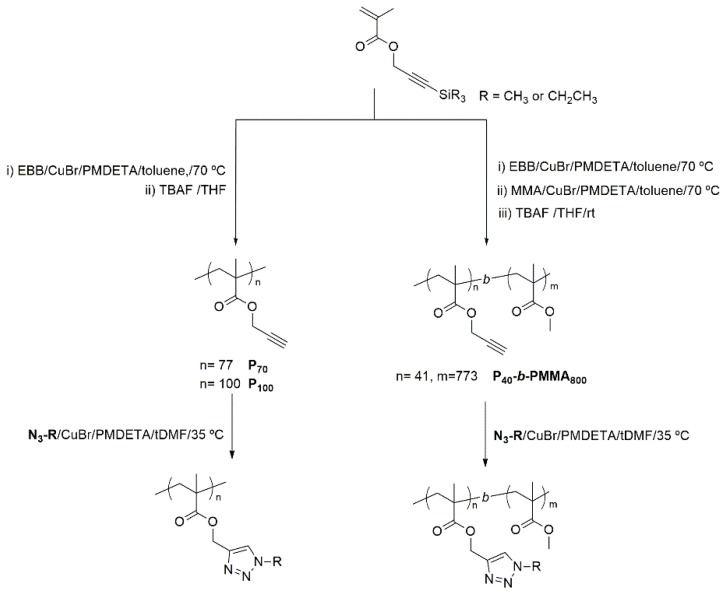
General outline for the synthesis of homopolymers and block copolymers.

**Figure 3:**
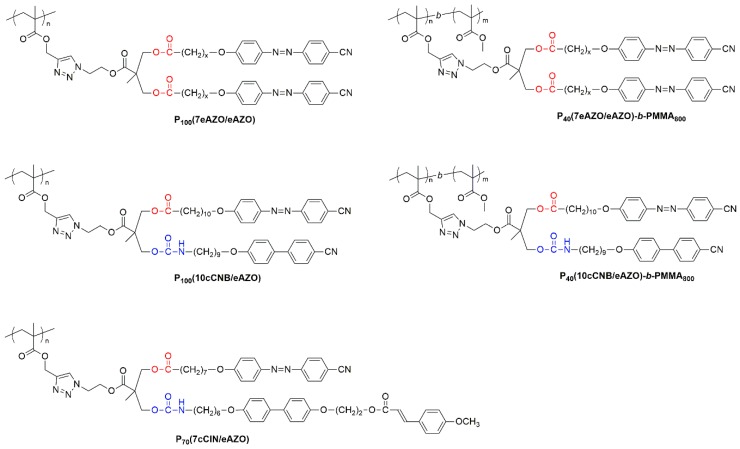
Chemical structure of homo- and block polymers discussed in this work.

Functionalized homopolymers were afforded by the postpolymerization modification of poly(propargyl methacrylate) with the described azides by CuAAC, whose viability and versatility has been previously recognized (Figure 2) [24,25,26]. Block copolymers were approached using a recently devised strategy based on the sequential ATRP polymerization of trimethylsilylpropargyl methacrylate and methyl methacrylate, and subsequent removal of the trimethylsilyl protecting groups [27]. Therefore, **P_40_TMS** was synthesized to be used as macroinitiator for the polymerization of **P_40_TMS-*b*-PMMA_800_** which, after quantitative removal of TMS protective groups, was functionalized by CuAAC (Figure 2). The chemical structures of the target polymers are collected in Figure 3, details of the synthesis are given in the Experimental Section and ^1^H NMR spectra are collected in the supporting information (Figures S1–S5).

Thermal stability of the polymers was studied by thermogravimetry (TGA) exhibiting a good thermal stability with major weight losses around 300 °C (Table 2). Block copolymers, **P_40_(7eAZO/eAZO)-*b*-PMMA_800_** and **P_40_(10cCNB/eAZO)-*b*-PMMA_800_**, suffered massive weight losses at temperatures slightly lower than their homopolymer counterparts, but still close to 300 °C.

**Figure 4:**
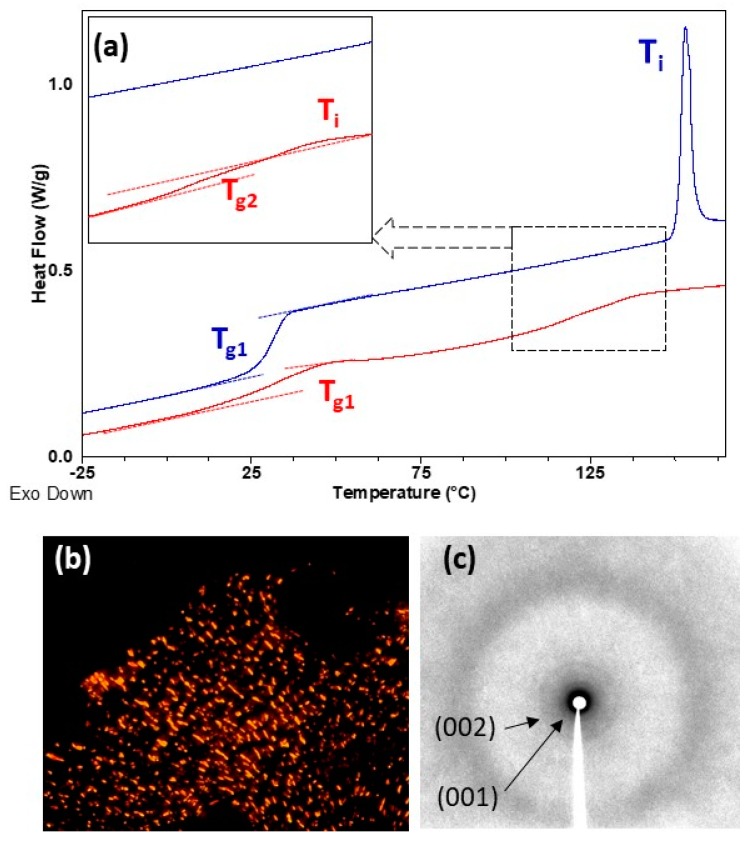
(**a**) DSC curves of **P_100_(7eAZO/eAZO)** (upper blue curve) and **P_40_(7eAZO/eAZO)-*b*-PMMA_800_** (lower red curve) registered during the second heating scan at 20 °C min^−1^. (**b**) POM photograph of **P_100_(7eAZO/eAZO)** taken at 145 °C on cooling from isotropic liquid. (**c**) XRD difractogram of **P_100_(7eAZO/eAZO)** registered at room temperature after annealing at 100 °C.

**Figure 5:**
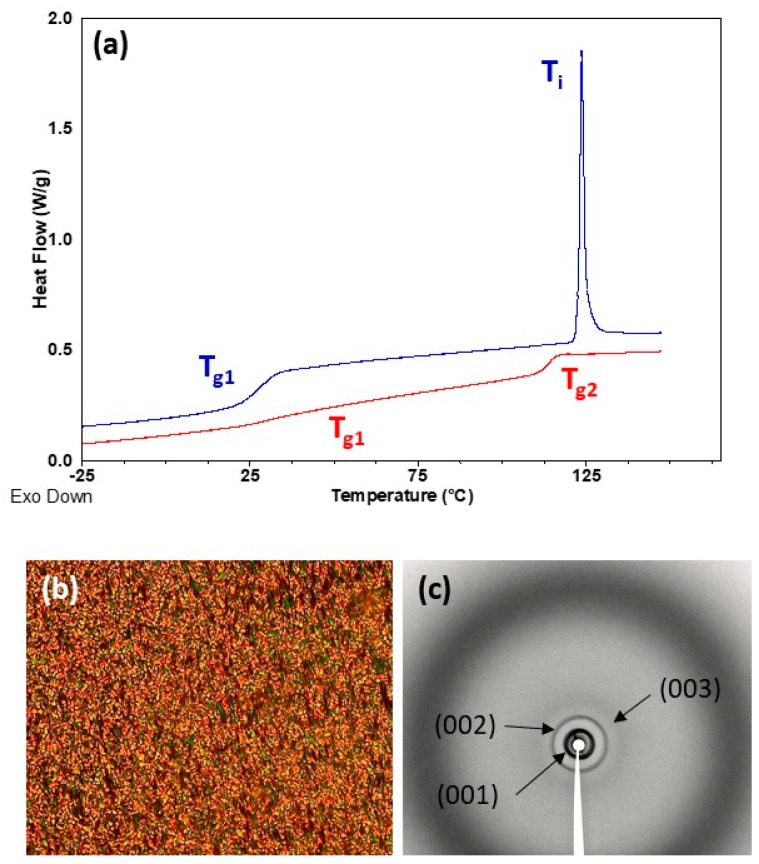
(**a**) DSC curves of **P_100_(10cCNB/eAZO)** (upper blue curve) and **P_40_(10cCNB/eAZO)-*b*-PMMA_800_** (lower red curve) registered during the second heating scan at 20 °C min^−1^. (**b**) POM photograph of **P_100_(10cCNB/eAZO)** taken at 105 °C on cooling from isotropic liquid. (**c**) XRD difractogram of **P_100_(10cCNB/eAZO)** registered at room temperature after annealing at 80 °C.

Thermal transitions were studied by DSC, POM and X-ray diffraction (XRD), and results are summarized in Table 2. DSC heating curves of all homopolymers presented a clear baseline jump corresponding to the glass transition (T_g_) followed by an endothermic peak associated to the mesophase-to-isotropic liquid transition (T_i_) (see Figure 4 and Figure 5). T_g_ values were just about room temperature for **P_100_(7eAZO/eAZO)** and **P_100_(10cCNB/eAZO)** at 34 and 27 °C respectively, and slightly higher for **P_70_(7cCIN/eAZO)**, 44 °C. Under POM, focal-conic textures coexisting with homeotropic areas were observed between T_g_ and T_i_ associated with the formation of a smectic A mesophase. Vitrification of the mesophase was observed upon cooling rather than crystallization. XRD patterns of vitrified samples registered at room temperature exhibited a diffuse ring at large angle corresponding to a distance of approx. 4.2 Å associated to the intermolecular distances between mesogenic units. Additionally, two sharp diffraction rings in the small angle regime were observed associated with the first and second order reflection of a lamellar phase. A layer spacing of 40–44 Å was measured that, considering the molecular length of the repeating unit estimated using Dreiding stereomodels, could be indicative of a smectic A with an interdigitated bilayered structure.

In POM, both block copolymers **P_40_(7eAZO/eAZO)-*b*-PMMA_800_** and **P_40_(10cCNB/eAZO)-*b*-PMMA_800_**, exhibited birefringent regions corresponding to a liquid crystalline phase, although not characteristic textures were observed even after prolonged annealing treatments (Figures S6a and S7a). Therefore, the mesophase was assessed by XRD using samples annealed at T = 0.9 × T_i_ for 30 min and quenched at room temperature. Diffraction patterns were essentially similar to those of the **P_100_(7eAZO/eAZO)** and **P_100_(10cCNB/eAZO)** homopolymers, consistent with a lamellar mesophase of a comparable layer spacing that could be presumably assigned to a smectic A mesophase (Figures S6b and S7b). DSC scans of block copolymers presented two glass transitions that matched reasonably well with that of the individual liquid crystalline homopolymer and PMMA, T_g1_ and T_g2_ respectively in Table 2 and Figure 4 and Figure 5. The same refers to T_i_. However, the associated ΔH_i_ was significantly depressed in comparison to the corresponding liquid crystalline homopolymers, which has been correlated to the disordering of the mesophase at the interface [30,31]. For **P_40_(7eAZO/eAZO)-*b*-PMMA_800_** a weak endothermic peak was observed (Figure 4) corresponding to T_i_ that in the case of **P_40_(10cCNB/eAZO)-*b*-PMMA_800_** was not detected (Figure 5) presumably masked by the glass transition of the PMMA block as they occur at essentially the same temperature range.

**Figure 6:**
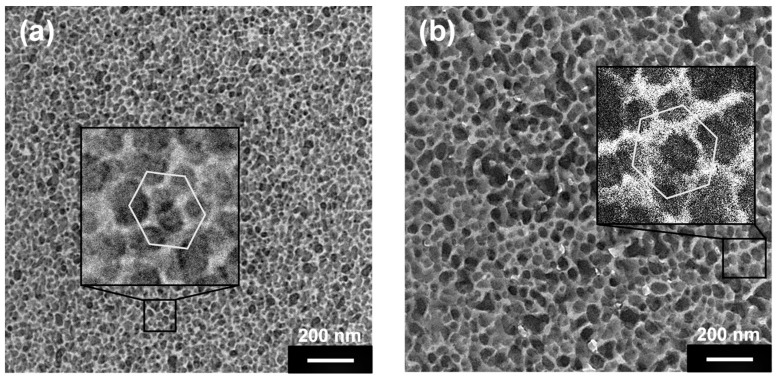
TEM images of films microtomed from small compact pellets thermally annealed at 150 °C for 24 h of block copolymers **P_40_(7eAZO/eAZO)-*b*-PMMA_800_** (**a**), and **P_40_(10cCNB/eAZO)-*b*-PMMA_800_** (**b**).

The ability of **P_40_(7eAZO/eAZO)-*b*-PMMA_800_** and **P_40_(10cCNB/eAZO)-*b*-PMMA_800_** block copolymers to segregate into phase separated microstructures was investigated by transmission electron microscopy (TEM). Thin films were microtomed from annealed polymer pellets embedded in an epoxy resin and then stained with ruthenium tetroxide. Images are collected in Figure 6 that demonstrated phase segregation between the two blocks being dark regions, stained with RuO_4_, azobenzene blocks into a bright unstained major phase corresponding to the PMMA matrix. However, the exact microphase separated structure was not obvious. Several factors affect the segregation of block copolymers, including the volume fraction of each block, the overall degree of polymerization and the mutual miscibility. In general, a lamellar morphology is usually prevalent at symmetric volume fractions, while spherical morphology might be predominant at highly asymmetric volume fractions. A moderate asymmetry might account for cylindrical or gyroid morphologies. Besides, the properties of the different blocks can modulate the segregation and it has been described that the segregation strength in side chain liquid crystal block copolymers is larger [32]. Schmidt and co-workers have described similar block copolymers in terms of composition whose TEM images resemble those obtained for **P_40_(7eAZO/eAZO)-*b*-PMMA_800_** and **P_40_(10cCNB/eAZO)-*b*-PMMA_800_**, which have a liquid crystalline mass fraction of 30 and 32%, respectively. They recognized a cylindrical morphology with dark round areas corresponding to cross sections of cylinders cut perpendicularly to the main axis [33]. Because in some regions it was possible to identify round areas in a roughly hexagonally packed pattern (see inset in Figure 6) and given the similarities between polymers, the morphology was tentatively assigned as cylindrical.

### 3.3. Chiral Induction and Stabilization of the Chiroptical Response

**Figure 7:**
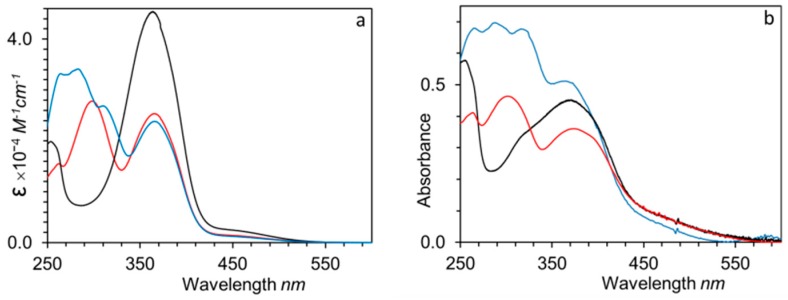
UV–Vis spectra in THF solution (**a**) and thin film (**b**) of **P_100_(7eAZO/eAZO)** (black lines), **P_100_(10cCNB/eAZO)** (red lines) and **P_70_(7cCIN/eAZO)** (blue lines).

UV–Vis spectra of homopolymers and copolymers were registered in THF solution (approx. 10^−5^ M of the repeating functional unit) and thin films (Figure 7 and Figure S8). In solution, **P_100_(7eAZO/eAZO)** and its block copolymer counterpart showed a main absorption band around 365 nm that corresponds to the π–π* transition of the *E*-4-cyanoazobenzene unit. The spectra of **P_100_(10cCNB/eAZO)** and its corresponding block copolymer displayed an additional band at 300 nm due to the π–π* transition of the 4-cyanobiphenyl unit. UV–Vis spectrum of **P_70_(7cCIN/eAZO)** combined the absorption bands of *E*-4-cyanoazobenzene (365 nm), *E*-4-methoxycinnamoyl (317 nm) and 4-ethoxy-4’-hexyloxybiphenyl (286 nm) units.

Thin films for optical characterization were prepared by casting chloroform solutions of the polymers onto clean fused silica slides. The films were annealed first at the isotropic state, then at the mesophase temperature, and finally quickly cooled to room temperature. UV–Vis spectra of the homopolymers and block copolymers thin films had about the same absorption maxima as those recorded in solution, although a slightly broadening of the bands was observed. In addition, cast films of the block copolymers exhibited a significant light scattering that can be attributed to the size of the segregated microdomains [34]. For **P_100_(7eAZO/eAZO)**, H-aggregation of chromophores was recognized by a shoulder at 326 nm. Likewise, the absorption maxima of the π–π* transition band of **P_40_(7eAZO/eAZO)-*b*-PMMA_800_** film was 25 nm shifted to shorter wavelengths compared to solution, which also points to H-aggregation of the azobenzene units, probably stimulated by the confinement of the chromophores into the block copolymer domains [27,35].

**Figure 8:**
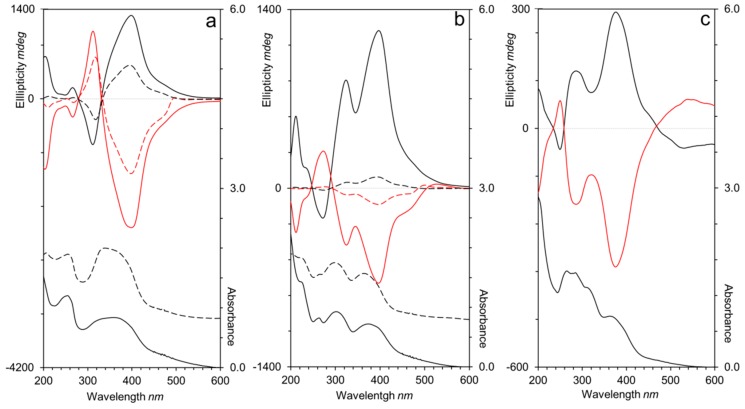
UV–Vis and electronic circular dichroism spectra of (**a**) **P_100_(7eAZO/eAZO)** and **P_40_(7eAZO/eAZO)-*b*-PMMA800**, (**b**) **P_100_(10cCNB/eAZO)** and **P_40_(10cCNB/eAZO)-*b*-PMMA800**, and (**c**) **P_70_(7cCIN/eAZO)** after irradiation with right (black) or left (red) handed CPL. Spectra of the homopolymers are displayed in solid lines and block copolymers in dotted lines.

After illumination with CPL of 488 nm at room temperature, homopolymers and block copolymers functionalized with AZO/AZO or CNB/AZO units exhibited electronic circular dichroism (ECD) spectra similar to those observed in previous work, with the sign dependent on the handedness of the excitation light [21,24,26,27]. The ECD spectra of irradiated AZO/AZO polymers films (Figure 8a) showed an exciton couplet corresponding to the π–π* *E*-azobenzene absorption band that confirmed the chiral organization of the azobenzene units. CPL irradiated films of CNB/AZO containing polymers (Figure 8b) showed a major band consisting of two overlapped exciton couplets that correspond to the π–π* transitions of 4-cyanobiphenyl and *E*-4-cyanoazobenzene units confirming the induction of a chiral arrangement of the non-photosensitive units by cooperative interactions with the photosensitive ones [24].

**Figure 9:**
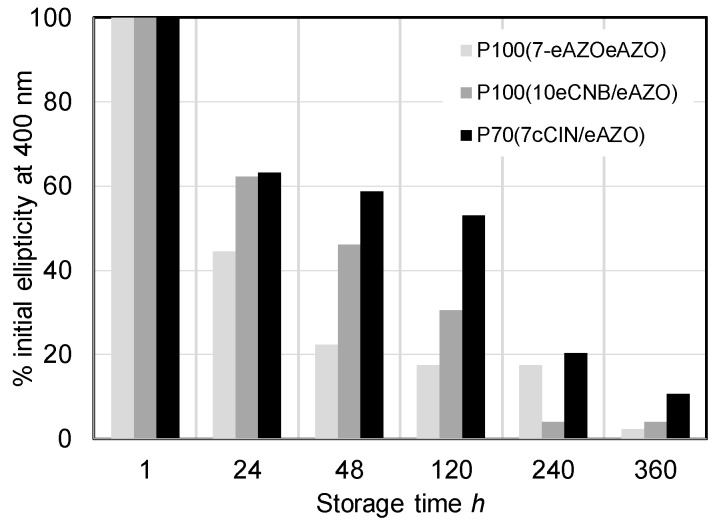
Stability of the photoinduced chirality of **P_100_(7eAZO/eAZO)**, **P_100_(10cCNB/eAZO)** and **P_70_(7cCIN/eAZO)**.

It should be noted that the photoinduced chiral signal in homopolymers **P_100_(7eAZO/eAZO)** and **P_100_(10cCNB/eAZO)** was remarkably more intense than the one detected in similar bifunctional homopolymers with either lower or higher T_g_ values [24,27]. Furthermore, **P_100_(10cCNB/eAZO)** showed a similar chiroptic response to that of **P_100_(7eAZO/eAZO)**, even though it has only one azobencene photoactive unit per repeating unit. Unfortunately, the T_g_ of both **P_100_(7eAZO/eAZO)** and **P_100_(10cCNB/eAZO)**, which was suitable to facilitate the transfer of chirality of light to the material, was not high enough to freeze the chiral order on the irradiated films when they were stored at room temperature. Consequently, the photoinduced chiral order was lost after approx. ten days (Figure 9). When these photoactive liquid crystalline polymers were integrated into a block copolymer, i.e., **P_40_(7eAZO/eAZO)-*b*-PMMA_800_** and **P_40_(10cCNB/eAZO)-*b*-PMMA_800_**, the photoinduced chiral signal was less intense than that of the corresponding homopolymers. Light dispersion of the incident beam due to the block copolymer microdomains might limit the transfer of chirality as it attenuates CPL intensity through the material. Nevertheless, the induced chiral response was maintained between 90%–100% of the initial value of the maximum ellipticity after storage at room temperature for at least six months. Likely, the restricted mobility of the photoactive moieties confined in the block copolymer microdomains should help to preserve the photoinduced chiral order compared to their respective homopolymers.

Irradiation of a **P_70_(7cCIN/eAZO)** film with CPL at 488 nm also led to the photoinduction of chirality as confirmed by the ECD bands associated with the π–π* transitions of both azobenzene and cinnamoyl units (Figure 8c). Since only the azobenzene unit is photoactive at 488 nm, the cooperative interactions between both functional units should be again responsible for the transference of chirality from the azobenzene to the cinnamoyl moieties. Although the higher T_g_ of **P_70_(7cCIN/eAZO)** compared to **P_100_(7eAZO/eAZO)** and **P_100_(10cCNB/eAZO)** delayed the loss of the photoinduced chiral signal during storage at room temperature, only 11% of the initial ellipticity at 400 nm was preserved after 15 days (Figure 9).

The main advantage of **P_70_(7cCIN/eAZO)** arises from the presence of two photosensitive moieties in the same repeating unit that provides a more complex response to light. Upon UV light irradiation, cinnamoyl units might undergo both *E-Z* photoisomerization and bimolecular [2+2] photocycloaddition. The relative occurrence extent of each reaction depends on the local concentration of the photochromic moiety but also on matrix effects, which determines the orientation and distance between adjacent cinnamoyl units. If [2+2] photocycloaddition prevails, as it is expected in condensed phases [36], a crosslinked polymer with a higher T_g_ could be generated, where the mobility of the polymeric chains should be consequently reduced and the temporal stability of the photoinduced chiral order improved. Therefore, a CPL irradiated **P_70_(7cCIN/eAZO)** film was exposed to UV light (260–350 nm) for different time intervals and the ECD and UV–Vis spectra were registered (Figure S9). A clear decrease of the absorbance and ellipticity of the π–π* transition of the cinnamoyl unit were observed on the UV-vis and ECD spectra respectively, confirming the occurrence of photoreactions. In addition, UV irradiation provoked a decrease of the chiral signal associated to π–π* azobenzene transition, mainly due to partial *E/Z* isomerization of the azobenzene moieties and/or the disturbance introduced by the [2+2] photocycloaddition adducts in the proximity of the azobenzene moieties. The optimum UV irradiation time was stablished at 1 h, which produces a crosslinked material insoluble in chloroform (the solvent used for film casting) that partially retains chiral organization of the azobenzene units. Because the photocrosslinking of cinnamoyl units reduces the mobility of polymer chains, the photocrosslinked material retained the photoinduced chiral organization for at least six months.

## 4. Conclusions

On the basis of minor structural changes, we have managed to get bifunctional liquid crystalline homopolymers with Tg values slightly above room temperature where a remarkably intense chiral signal was photoinduced using CPL. In contrast to previous examples [15] the intensity of the signal was maintained even if one of the two photoaddressable 4-cyanoazobenzenes of the repeating unit was replaced by a photoinert 4-cyanobiphenyl. The incorporation of these photoaddressable liquid crystalline polymers into diblock copolymers restrains the mobility of the photoactive nanodomains, improving the stability of the photoinduced chiral order, which is otherwise lost after a few days at room temperature.

Alternatively, combining both azobenzene and cinnamate moieties into the same repeating unit allows for an efficient chirality induction and stabilization of the chiral signal by using an orthogonal irradiation procedure that combines visible CPL and UV light.

## Figures and Tables

**Table 1:** Transition Temperatures of Azides.

Azide	Transitions Temperatures (°C) [ΔH (kJ mol^−1^)] ^1^
**N_3_–7eAZO/eAZO**	C 51 [26.2] SmA 80 N 82 I ^2^
**N_3_–10cCNB/eAZO**	g −2 SmA 72 [7.0] I
**N_3_–7cCIN/eAZO**	g 21 SmA 48 [7.6] I

^1^ Temperatures and associated enthalpies (in square brackets) determined by DSC at 10 °C min^−1^ rate from the second heating scan. C: crystal; g: glass; SmA: smectic A; N: nematic; I: isotropic liquid. ^2^ Peaks corresponding to SmA-to-N and N-to-I transitions are overlapped with a whole enthalpy value of 5.0 kJ mol^−1^.

**Table 2:** Thermal Stability and Transition Temperatures of Polymers.

Polymer	TGA (°C) ^1^	T_g1_ (°C) ^2^	T_g2_ (°C) ^2^	T_i_ (°C) [ΔH_i_ (kJ mol^−^^1^)] ^3^	Mesophase	*d* (Å) ^4^
**P_100_(7eAZO/eAZO)**	320	34	–	153 [6.4]	SmA	40
**P_100_(10cCNB/eAZO)**	313	27	–	124 [7.6]	SmA	44
**P_70_(7cCIN/eAZO)**	319	44	–	101 [5.3]	SmA	45
**P_40_(7eAZO/eAZO)-*b*-PMMA_800_**	305	34	117	137 [3.4]	Sm ^6^	39
**P_40_(10cCNB/eAZO)-*b*-PMMA_800_**	297	31	114	120 ^5^	Sm ^6^	45

^1^ Decomposition temperature determined by TGA given at the onset of the weight loss curve. ^2^ Glass transition temperatures (T_g_) determined by DSC during the second heating scan (for homopolymers) or the first heating scan (for block copolymers) at 20 °C min^−1^. T_g1_ refers to the liquid crystalline block and T_g2_ refers to the PMMA block. ^3^ Mesophase-to-isotropic liquid transition temperature (T_i_) detected on the heating scan at 20 °C min^−1^. Associated enthalpy values are shown in square brackets and giving in kJ per mole of repeating unit referred to the liquid crystalline block. ^4^ Layer spacing determined by XRD. ^5^ Temperature determined by POM. T_i_ overlapped with T_g2_ on the DSC curve, enthalpy could not be accurately calculated. ^6^ Not identified smectic phase.

## Supplementary Materials

The following are available online at https://www.mdpi.com/2073-4360/11/5/885/s1, Synthesis and characterization of azides. Figures S1–S5: ^1^H NMR (400 MHz, CDCl_3_) spectrum of **P_100_(7eAZO/eAZO)**, **P_100_(10cCNB/eAZO)**, **P_70_(7cCIN/eAZO)**, **P_40_(7eAZO/eAZO)-*b*-PMMA_800_** and **P_40_(9cCNB/eAZO)-*b*-PMMA_800_**, Figure S6: (a) POM photograph taken at 125 °C on cooling from isotropic liquid and (b) XRD difractogram registered at room temperature after annealing at 96 °C of **P_40_(7eAZO/eAZO)-*b*-PMMA_800_**, Figure S7: (a) POM photograph taken at 105 °C on cooling from isotropic liquid and (b) XRD difractogram registered at room temperature after annealing at 80 °C of **P_40_(10cCNB/eAZO)-*b*-PMMA_800_**, Figure S8: UV-vis spectra in THF solution and thin film of **P_40_(7eAZO/eAZO)-*b*-PMMA_800_** and **P_40_(10cCNB/eAZO)-*b*-PMMA_800_**, Figure S9: UV-vis and ECD spectra of **P_70_(7cCIN/eAZO)** film after r-CPL irradiation and r-CPL irradiated film exposed at UV light for 1 min, 1 h, 2 h and 3 h, Figure S10: Scheme of the experimental set-up used for CPL irradiation experiments.

## References

[1] Natansohn A., Rochon P. (2002). Photoinduced Motions in Azo-Containing Polymers. Chem. Rev..

[2] Zola R.S., Bisoyi H.K., Wang H., Urbas A.M., Bunning T.J., Li Q. (2019). Dynamic control of light direction enabled by stimuli-responsive liquid crystal gratings. Adv. Mater..

[3] Bisoyi H.K., Li Q. (2016). Light-driven liquid crystalline materials: From photo-induced phase transitions and property modulations to applications. Chem. Rev..

[4] Shibaev V.P., Bobrovsky A.Y. (2017). Liquid crystalline polymers: Development trends and photocontrollable materials. Russ. Chem. Rev..

[5] Blasco E., Piñol M., Berges C., Sánchez-Somolinos C., Oriol L., Aguilar M.R., Román J.S. (2014). Smart Polymers and Their Applications.

[6] Yu H. (2014). Recent advances in photoresponsive liquid crystalline polymers containing azobenzene chromophores. J. Mater. Chem. C.

[7] Shishido A. (2010). Rewritable holograms based on azobenzene-containing liquid-crystalline polymers. Polym. J..

[8] Shibaev V., Bobrovsky A., Boiko N. (2003). Photoactive liquid crystalline polymer systems with light-controllable structure and optical properties. Prog. Polym. Sci..

[9] Kawatsuki N., Goto K., Kawakami T., Yamamoto T. (2002). Reversion of alignment direction in the thermally enhanced photoorientation of photo-cross-linkable polymer liquid crystal films. Macromolecules.

[10] Kawatsuki N., Tsutsumi R., Takatsuka H., Sakai T. (2007). Influence of alkylene spacer length on thermal enhancement of photoinduced optical anisotropy in photo-cross-linkable liquid crystalline polymeric films and their composites with non-liquid-crystalline monomers. Macromolecules.

[11] Zanutta A., Colella L., Bertarelli C., Bianco A. (2013). Understanding the mechanism of refractive index modulation in materials undergoing photo-Fries rearrangement. Opt. Mater..

[12] Kawatsuki N., Suehiro C., Yamamoto T. (1998). Photoinduced alignment of photo-cross-linkable side-chain liquid crystalline copolymers comprising cinnamoylethoxybiphenyl and cyanobiphenyl groups. Macromolecules.

[13] Kawatsuki N., Uchida E., Yamamoto T. (2003). Photocontrol of birefringence and in-plane molecular orientation in copolymer liquid crystal films with 4-methoxyazobenzene and photo-cross-linkable side groups. Macromol. Chem. Phys..

[14] Li X., Cui J., Zhang W., Huang J., Li W., Lin C., Jiang Y., Zhang X., Li G. (2011). Controllable photo-switching of cinnamate-based photonic films with remarkable stability. J. Mater. Chem..

[15] Yu H. (2014). Photoresponsive liquid crystalline block copolymers: From photonics to nanotechnology. Prog. Polym. Sci..

[16] Sano M., Hara M., Nagano S., Shinohara Y., Amemiya Y., Seki T. (2015). New aspects for the hierarchical cooperative motions in photoalignment process of liquid crystalline block copolymer films. Macromolecules.

[17] Sano M., Shan F., Hara M., Nagano S., Shinohara Y., Amemiya Y., Seki T. (2015). Dynamic photoinduced realignment processes in photoresponsive block copolymer films: Effects of the chain length and block copolymer architecture. Soft Matter.

[18] Yu H., Asaoka S., Shishido A., Iyoda T., Ikeda T. (2007). Photoinduced nanoscale cooperative motion in a well-defined triblock copolymer. Small.

[19] Tong X., Cui L., Zhao Y. (2004). Confinement effects on photoalignment, photochemical phase transition, and thermochromic behavior of liquid crystalline azobenzene-containing diblock copolymers. Macromolecules.

[20] Nikolova L., Todorov T., Ivanov M., Andruzzi F., Hvilsted S., Ramanujam P.S. (1997). Photoinduced circular anisotropy in side-chain azobenzene polyesters. Opt. Mater..

[21] Tejedor R.M., Millaruelo M., Oriol L., Serrano J.L., Alcala R., Rodrıguez F.J., Villacampa B. (2006). Photoinduced supramolecular chirality in side-chain liquid crystalline azopolymers. J. Mater. Chem..

[22] Benelli T., Lanzi M., Mazzocchetti L., Giorgini L. (2017). Chirality on amorphous high-Tg polymeric nanofilms: Optical activity amplification by thermal annealing. Nanomaterials.

[23] Yue Y., Norikane Y., Azumi R., Koyama E. (2018). Light-induced mechanical response in crosslinked liquid-crystalline polymers with photoswitchable glass transition temperatures. Nat. Commun..

[24] Royes J., Rebole J., Custardoy L., Gimeno N., Oriol L., Tejedor R.M., Piñol M. (2012). Preparation of side-chain liquid crystalline azopolymers by CuAAC postfunctionalization using bifunctional azides: Induction of chirality using circularly polarized light. J. Polym. Sci. Part A Polym. Chem..

[25] Royes J., Provenzano C., Pagliusi P., Tejedor R.M., Piñol M., Oriol L. (2014). A bifunctional amorphous polymer exhibiting equal linear and circular photoinduced birefringence. Macromol. Rapid Commun..

[26] Roche A., García-Juan H., Royes J., Oriol L., Piñol M., Audia B., Pagliusi P., Provenzano C., Cipparrone G. (2018). Tuning the thermal properties of azopolymers synthesized by post-functionalization of poly(propargyl methacrylate).with azobenzene azides: Influence on the generation of linear and circular birefringences. Macromol. Chem. Phys..

[27] Royes J., Nogales A., Ezquerra T.A., Oriol L., Tejedor R.M., Piñol M. (2018). Effect of the polymer architecture on the photoinduction of stable chiral organizations. Polymer.

[28] Ladmiral V., Mantovani G., Clarkson G.J., Cauet S., Irwin J.L., Haddleton D.M. (2006). Synthesis of neoglycopolymers by a combination of “click chemistry” and living radical polymerization. J. Am. Chem. Soc..

[29] Goodwin A.P., Lam S.S., Fréchet J.M.J. (2007). Rapid, efficient synthesis of heterobifunctional biodegradable dendrimers. J. Am. Chem. Soc..

[30] Tian Y., Watanabe K., Kong X., Abe J., Iyoda T. (2002). Synthesis, nanostructures, and functionality of amphiphilic liquid crystalline block copolymers with azobenzene moieties. Macromolecules.

[31] Yamada M., Itoh T., Nakagawa R., Hirao A., Nakahama S., Watanabe J. (1999). Synthesis of side-chain liquid crystalline homopolymers and block copolymers with cyanobiphenyl moieties as the mesogen by living anionic polymerization and their thermotropic phase behavior. Macromolecules.

[32] Hamley I.W., Castelletto V., Lu Z.B., Imrie C.T., Itoh T., Al-Hussein M. (2004). Interplay between smectic ordering and microphase separation in a series of side-group liquid-crystal block copolymers. Macromolecules.

[33] Breiner T., Kreger K., Hagen R., Häckel M., Kador L., Müller A.H.E., Kramer E.J., Schmidt H.-W. (2007). Blends of poly(methacrylate) block copolymers with photoaddressable segments. Macromolecules.

[34] Lodge T. (1994). Characterization of polymer materials by scattering techniques, with applications to block copolymers. Microchim. Acta.

[35] Morikawa Y., Nagano S., Watanabe K., Kamata K., Iyoda T., Seki T. (2006). Optical alignment and patterning of nanoscale microdomains in a block copolymer thin film. Adv. Mater..

[36] Oriol L., Piñol M., Serrano J.L., Tejedor R.M. (2003). Synthesis, characterization and photoreactivity of liquid crystalline cinnamates. J. Photochem. Photobiol. A Chem..

