# Supplementary material for: Strategies to Stabilize the Photoinduced Supramolecular Chirality in Azobenzene Liquid Crystalline Polymers
Source: Polymers (Basel). 2019 May 15;11(5):885. doi: 10.3390/polym11050885 (PMC6572559; doi:10.3390/polym11050885)
Supplement: Supplementary file 1 [file polymers-11-00885-s001.pdf]

Article

Strategies to stabilize the photoinduced supramolecular chirality in azobenzene liquid crystalline polymers

Jorge Royes [1,2], Luis Oriol [1], Rosa M. Tejedor[1,3,*] and Milagros Piñol [1,*]

[1] Departamento de Química Orgánica, Instituto de Ciencia de Materiales de Aragón (ICMA), Universidad de Zaragoza-CSIC, c/ Pedro Cerbuna 12, 50009 Zaragoza, Spain
[2] Present address: UMR 8640, CNRS, École Normale Superieure Département de Chimie, 24 rue Lhomond, 75005, Paris, France
[3] Centro Universitario de la Defensa, Academia General Militar, Ctra. de Huesca s/n, 50090 Zaragoza, Spain
* Correspondence: rtejedor@unizar.es (R.M.T.) mpinol@unizar.es (M.P.)

Received: date; Accepted: date; Published: date

1. Synthesis and characterization of azides

Azide **N_3-7eAZO/eAZO** was synthesized following a previously reported procedure [1]. **N_3-10cCNB/eAZO** and **N_3-10cCIN/eAZO** were preparared according to procedures described for homologous compounds [2].

1.2. Synthesis of the azide N_3-7eAZO/eAZO

Azide **N_3-7eAZO/eAZO** was synthesized as shown in Scheme S1 by esterification of the hydroxyl groups of azide (**N_3-bisMPA**) with the acyl choride of 4-(4'-cyanophenylazophenyloxy)octanoic acid.

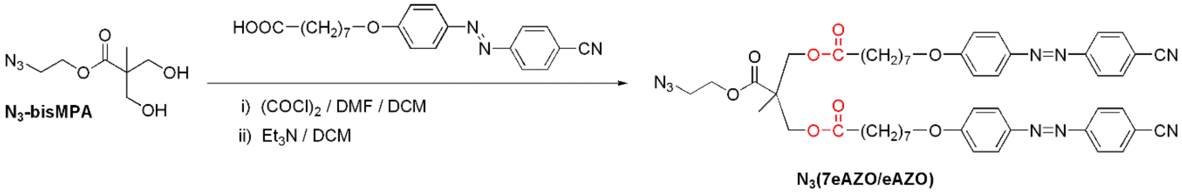

Scheme S1. Synthesis of the azide **N_3-7eAZO/eAZO**

Oxalyl chloride (1.18 mL, 13.7 mmol) was added dropwise over a solution of 4-(4'-cyanophenylazophenyloxy)octanoic acid (2.5 g, 6.8 mmol) in *N,N*-dimethylformamide (DMF) (100 μL) and dried dichloromethane (DCM) (50 mL) at 0 °C under argon atmosphere. After stirring for 4 h at room temperature, the solvent was removed under reduced pressure. This acyl chloride was added over a solution of **N_3-bisMPA** (0.630 g, 3.2 mmol) and triethylamine (1.78 mL, 12.4 mmol) in dried dichloromethane (80 mL) immersed into an ice-salt bath and kept under argon atmosphere. The reaction was stirred overnight at room temperature before adding a 10% w/w aqueous solution of NH_4Cl (aq) (160 mL). The organic phase was separated and the aqueous one extracted with dichloromethane (3×50 mL). The combined organic fractions were dried over anhydrous $MgSO_4$ and evaporated to dryness. The residue was purified by medium pressure liquid chromatography (MPLC) using silica gel (60Å, 60-120 mesh) and eluting first with dichloromethane and gradually

increasing the polarity of the eluent up to dichloromethane/ethyl acetate (9:1). N_3-7eAZO/eAZO was finally recrystallized from ethanol and isolated in 58% yield. IR (KBr disk, cm^{-1}): 2223 (C≡N), 2094 (N_3), 1734 (C=O). ^{1}H NMR(400 MHz, $CDCl_3$, δ, ppm): 8.02 – 7.85 (m, 8H), 7.85 – 7.70 (m, 4H), 7.08–6.90 (m, 4H), 4.37–4.17 (m, 6H), 4.04 (t, *J* = 6.4 Hz, 4H), 3.49 (t, J = 5.3 Hz, 2H), 2.33 (t, *J* = 7.5 Hz, 4H), 1.90–1.75 (m, 4H), 1.70–1.59 (m, 4H), 1.54–1.25 (m, 15H). ^{13}C NMR (100 MHz, $CDCl_3$, δ,ppm): 173.05, 172.51, 162.64, 154.72, 146.64, 133.08, 125.40, 123.00, 118.69, 114.81, 113.07, 68.31, 65.13, 63.70, 49.63, 46.42, 33.97, 29.01, 28.93, 25.77, 24.71, 17.66. HRMS (ESI+) *m/z* Exp.: (calc. for $C_{49}H_{55}N_9O_8Na^+$) 920.4039 (920.4071). Elem. Anal. Exp. (calc. for $C_{49}H_{55}N_9O_8$): C 65.35% (65.54%), H 5.89% (6.17%), N 13.74% (14.04%).

1.2. Synthesis of the azide N_3-10cCNB/eAZO

Azide **N_3-10cAZO/eAZO** was synthesized from a cyclic carbonate **N_3-bisMPA-carbonate** by reacting sequentially the amine 4-cyano-4'-(9-aminononyloxy)biphenyl **(3)** and 4-(4'-cyanophenylazophenyloxy)undecanoic acid prepared following a reported procedure (Scheme S2).

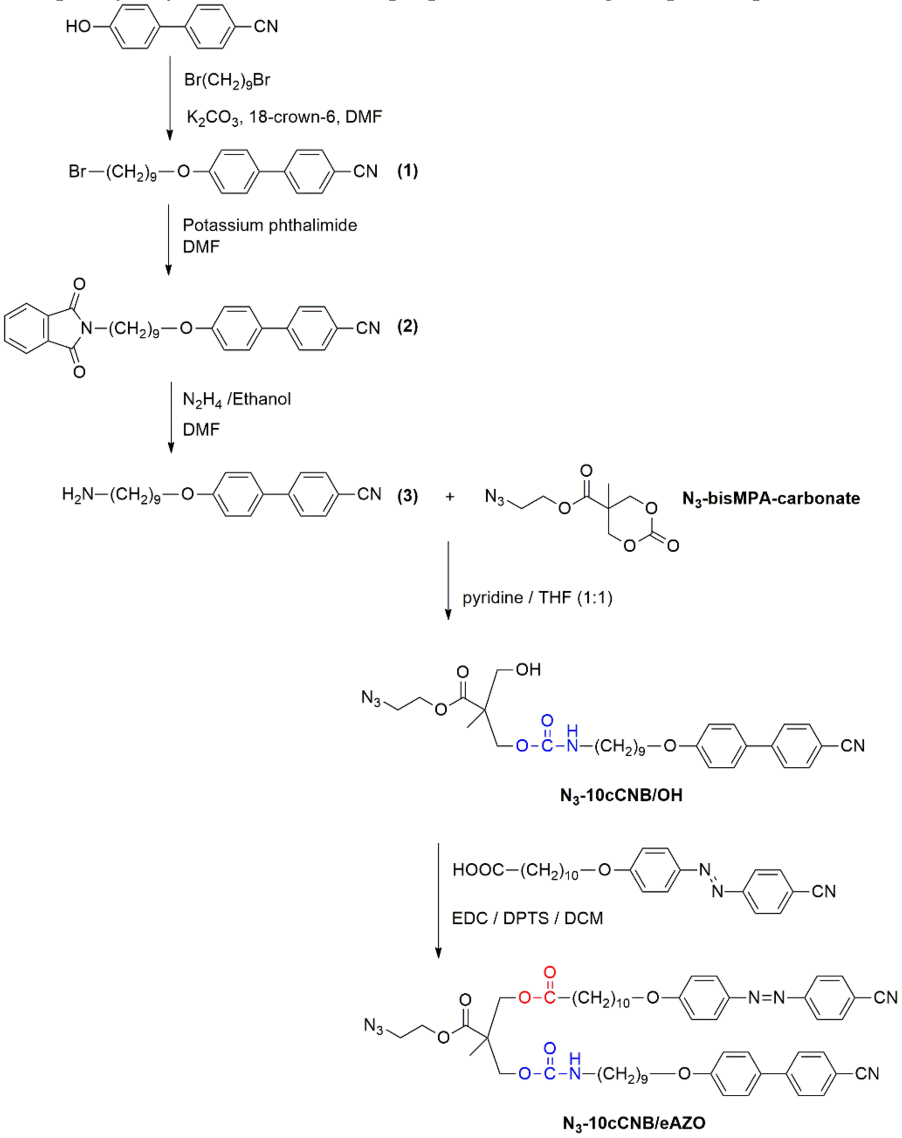

Scheme S2. Synthesis of the azide **N_3-10cCNB/eAZO**

Synthesis of 4'-((9-bromononyl)oxy)-[1,1'-biphenyl]-4-carbonitrile (1). A solution of 4-cyano-4′-hydroxybiphenyl (4.881 g, 25.0 mmol) in DMF (25 mL) was dropwise added over a suspension of 1,9-dibromononane (8.582 g, 30.0 mmol), 18-crown-6 (0.360 g, 1.3 mmol) and K_2CO_3 (6.911 g, 50.0 mmol) in DMF (10 mL). The reaction was heated at 120ºC for 24 h and then water was added (70 mL). The reaction was extracted with hexane/ethyl acetate (1:1) (3×70 mL) and the combined organic extracts washed with water (2×100 mL) and saturated NaCl (1×100 mL), dried over anhydrous $MgSO_4$ and finally evaporated to dryness. The crude was purified first by flash column chromatography on silica gel using DCM as eluent and recrystallized from ethanol to yield the target bromide compound (1) (52% yield). IR (KBr disk, cm^{-1}): 2225 (C≡N), 532 (C–Br). ^{1}H NMR (400 MHz, $CDCl_3$, δ, ppm): 7.73–7.60 (m, 4H), 7.57–7.49 (m, 2H), 7.05–6.95 (m, 2H), 4.17 (t, J = 6.5 Hz, 2H), 4.01 (t, J = 6.5 Hz, 2H), 1.91–1.75 (m, 2H), 1.71–1.60 (m, 2H), 1.51–1.43 (m, 2H), 1.43–1.23 (m, 8H). ^{13}C NMR(100 MHz, $CDCl_3$, δ, ppm): 159.80, 145.27, 132.55, 131.31, 128.49, 128.31, 127.07, 119.08, 115.35, 115.10, 110.09, 68.15, 64.06, 29.29, 29.22, 28.50, 26.02, 25.79.

Synthesis of 4'-((9-(1,3-dioxoisoindolin-2-yl)nonyl)oxy)-[1,1'-biphenyl]-4-carbonitrile (2). A solution of potassium phthalimide (1.822 g, 10.0 mmol) in DMF (4 mL) was dropwise added over bromide compound (1) (2.000 g, 5.0 mmol) in DMF (6 mL). The reaction was stirred at 180 ºC for 24 h, then cooled to room temperature and poured into water (40 mL). The mixture was extracted with DCM (3×40 mL) and the combined organic extracts washed with water (2×120 mL), saturated NH_4Cl aqueous solution (120 mL), saturated NaCl aqueous solution (120 mL), and finally dried over anhydrous $MgSO_4$ and evaporated to dryness. The crude was recrystallized from ethanol/water to yield the phthalimide compound (2) as white crystals (96 % yield). IR (KBr disk, cm^{-1}): 2218 (C≡N), 1770 (C=O). ^{1}H NMR (400 MHz, $CDCl_3$, δ, ppm): 7.87–7.80 (m, 2H), 7.74–7.66 (m, 4H), 7.66 - 7.60 (m, 2H), 7.55–7.49 (m, 2H), 7.00–6.96 (m, 2H), 4.06–3.95 (m, 2H), 3.72–3.61 (m, 2H), 1.85–1.74 (m, 2H), 1.71–1.62 (m, 2H), 1.52–1.23 (m, 10H). ^{13}C NMR (100 MHz, $CDCl_3$, δ, ppm): 168.47, 159.84, 145.33, 133.79, 132.61, 132.18, 131.26, 128.32, 127.17, 123,14, 119,13, 115,12, 110,07, 68,11, 38,03, 29,36, 29,32, 29,22, 29,08, 28,60, 26,81, 26,03.

Synthesis of 4-cyano-4'-(9-aminononyloxy)biphenyl (3). Hydrazine monohydrate (0.4 mL, 8.0 mmol) was added over a solution of the phthalimide (2) (2.500 g, 5.36 mmol) in absolute ethanol (125 mL) and the mixture stirred at reflux temperature for 3 h. Once the reaction was cooled to room temperature and the white precipitate discarded out, the ethanolic solution was evaporated to dryness to render the target amine (3) as a white powder that was washed with hexane (quantitative yield). IR (KBr disk, cm^{-1}): 3473, 3349 (N–H), 2224 (C≡N), 1603. ^{1}H NMR (400 MHz, $CDCl_3$, δ, ppm): 7.71–7.66 (m, 2H), 7.66–7.61 (m, 2H), 7.55–7.48 (m, 2H), 7.03–6.94 (m, 2H), 4.00 (t, J = 6.5 Hz, 2H), 2.69 (t, J = 7.0 Hz, 2H), 1.95 (broad s, 2H), 1.86–1.73 (m, 2H), 1.53–1.41 (m, 4H), 1.40–1.23 (m, 8H). ^{13}C NMR (100 MHz, $CDCl_3$, δ, ppm): 159.76, 145.26, 132.53, 131.23, 128.29, 127.04, 119.09, 115.05, 110.00, 68.13, 42.05, 33.43, 29.50, 29.38, 29.30, 29.19, 26.84, 26.00.

Synthesis of **N_3-10cCNB/OH**. A dispersion of the amine (3) (1.514 g, 4.5 mmol) in anhydrous THF/pyridine (5 mL/10 mL) was added dropwise to a solution of the cyclic carbonate N_3-bisMPA-carbonate (1.031 g, 4.5 mmol) and pyridine (6 mL) in anhydrous THF/pyridine (10 mL/5 mL) under argon atmosphere. The reaction mixture was stirred at room temperature for 18 h. Then, ethyl acetate (150 mL) was added and the resulting solution washed with $NaHSO_4$ 1M (2×90 mL), saturated aqueous NaCl solution (60 mL) and dried over anhydrous $MgSO_4$. The volatiles were removed in a rotary-evaporator and the crude recrystallized from abs. ethanol to give compound N_3-10cCNB/OH as a white solid (65% yield). IR (KBr disk, cm^{-1}): 3474 (O–H), 3393 (N–H), 2233 (C≡N), 2108 (N_3), 1713 (C=O). ^{1}H NMR (400 MHz, $CDCl_3$, δ, ppm): 7.72–7.60 (m, 4H), 7.56–7.49 (m, 2H), 7.03–6.94 (m, 2H), 4.80 (t, J = 5.8 Hz, 1H), 4.35 (d, J_{gem} = 11.6 Hz, 1H), 4.33–4.26 (m, 2H), 4.23 (d, J_{gem} = 11.6 Hz, 1H), 4.00 (t, J = 6.5 Hz, 2H), 3.70 (d, J_{gem} = 11.8 Hz, 1H), 3.62 (d, J_{gem} = 11.8 Hz, 1H), 3.49 (t, J = 5.2 Hz, 2H), 3.17 (dt, J = 6.7, 6.7 Hz, 2H), 3.12 (broad s, 1H), 1.86–1.73 (m, 2H), 1.53–1.42 (m, 4H), 1.39–1.27 (m,

8H), 1.22 (s, 3H). ^{13}C NMR (100 MHz, $CDCl_3$, δ,ppm): 174.20, 159.76, 156.91, 145.26, 132.54, 131.25, 128.30, 127.05, 119.10, 115.06, 110.01, 68.11, 65.51, 64.06, 63.45, 49.79, 48.91, 41.17, 29.81, 29.41, 29.26, 29.18, 29.14, 26.65, 25.98, 17.46.

Synthesis of **N_3-10cCNB/eAZO**. A solution of the intermediate azide **N_3-10cCNB/OH** (1.527 g, 2.7 mmol), 4-(4′-cyanophenylazophenyloxy)undecanoic acid (1.304 g, 3.2 mmol) and 1,4-dimethylpyridinium *p*-toluenesulfonate (DPTS) (0.143 g, 0.5 mmol) in anhydrous DCM (30 mL) was cooled with an ice-salt bath under argon atmosphere for 20 min. Then a dispersion of 1-ethyl-3-(3-dimethylaminopropyl)carbodiimide (EDC) (0.621 g, 3.3 mmol) in anhydrous DCM (20 mL) was added dropwise. Once addition was completed, the reaction mixture was allowed to warm to room temperature and stirred for 24 h while the reaction was monitored by thin layer chromatography using DCM/ethyl acetate (9:1) as eluent. The reaction mixture was diluted with dichloromethane (200 mL) and washed with distilled water (2×200 mL) and saturated aqueous NaCl solution (200 mL), and dried over $MgSO_4$. The volatiles were removed and the solid obtained was purified by medium pressure liquid chromatography (MPLC) using silica gel (60Å, 60-120 mesh) and dichloromethane/ethyl acetate (9:1) as eluent. Subsequent recrystallization in absolute ethanol yielded the target N_3-10cCNB/eAZO as an orange powder (86% yield). IR (KBr disk, cm^{-1}): 3382 (N–H), 2226 (C≡N), 2106 (N_3), 1737 (C=O). ^1H NMR (400 MHz, $CDCl_3$, δ, ppm): 7.97–7.87 (m, 4H), 7.82–7.76 (m, 2H), 7.71–7.66 (m, 2H), 7.66–7.60 (m, 2H), 7.55–7.48 (m, 2H), 7.04–6.95 (m, 4H), 4.72 (t, *J* = 6.7 Hz, 1H), 4.33–4.18 (m, 6H), 4.05 (t, *J* = 6.5 Hz, 2H), 3.99 (t, *J* = 6.5 Hz, 2H), 3.48 (t, *J* = 5.5 Hz, 2H), 3.15 (td, *J* = 6.3, 5.5 Hz, 2H), 2.31 (t, *J* = 7.6 Hz, 2H), 1.90–1.73 (m, 4H), 1.54–1.41 (m, 6H), 1.41–1.20 (m, 23H). ^{13}C NMR (100 MHz, $CDCl_3$, δ,ppm): 173.30, 172.79, 162.72, 159.75, 155.77, 154.77, 146.65, 145.24, 133.13, 132.53, 131.23, 128.29, 127.03, 125.44, 123.03, 119.09, 118.65, 115.04, 114.85, 113.08, 110.00, 68.45, 68.10, 65.81, 65.23, 63.64, 49.67, 46.71, 41.08, 34.08, 29.85, 29.46, 29.42, 29.35, 29.31, 29.20, 29.17, 29.07, 26.68, 25.98, 25.96, 24.83, 17.53. HRMS (ESI+) *m/z* Exp.: (calc. for $C_{54}H_{66}N_8O_8Na^+$) 977.4629 (977.4896). Elem. Anal. Exp. (calc. for $C_{54}H_{66}N_8O_8$): C 67.95% (67.90%), H 7.01% (6.96%), N 11.66% (11.73%).

1.3. Synthesis of the azide N_3-7cCIN/eAZO

The azide **N_3-7cCIN/eAZO** was prepared following the synthetic scheme presented in Scheme S3.

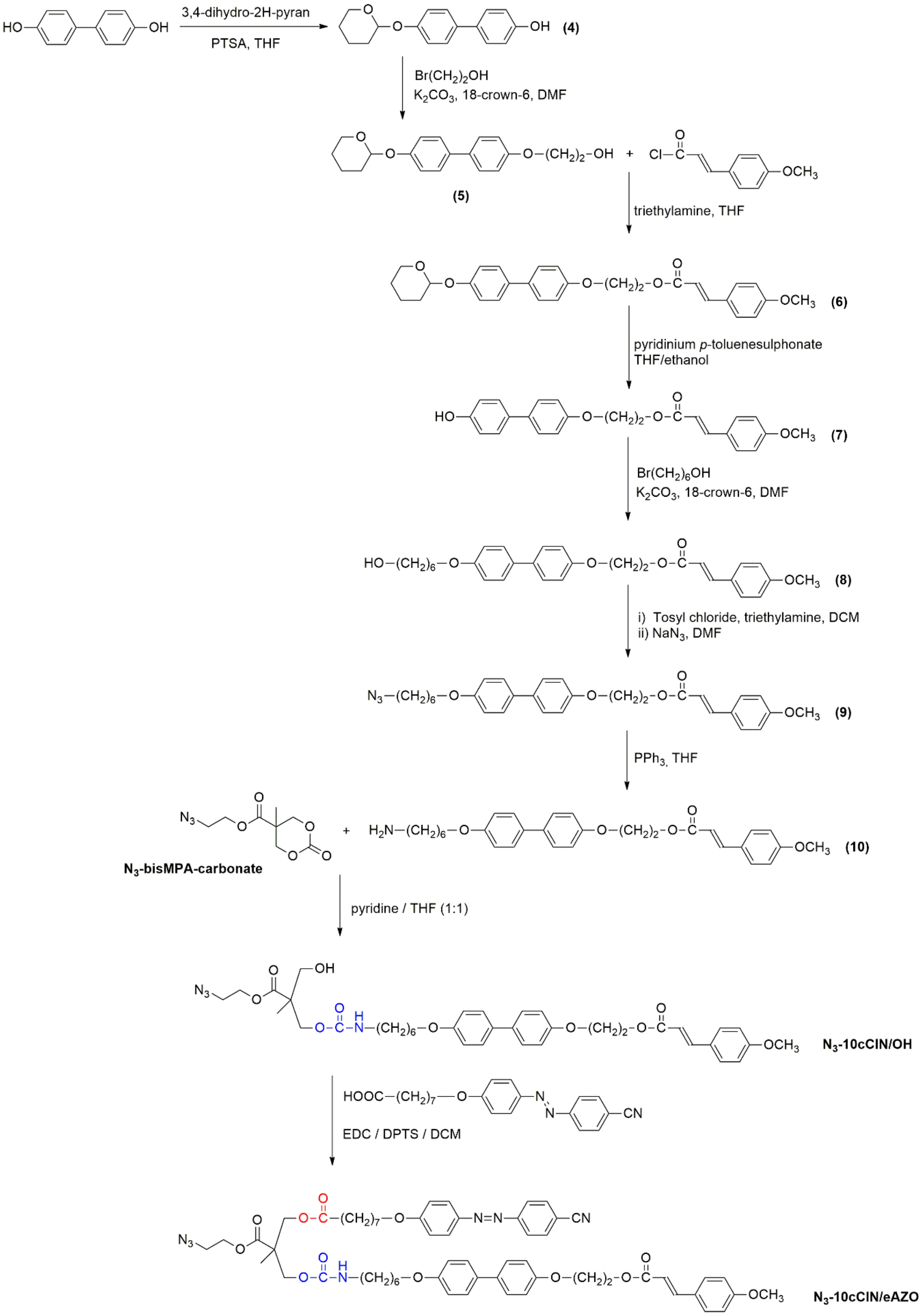

Scheme S3. Synthesis of the azide **N_3-7cCIN/eAZO**

Synthesis of 4'-((tetrahydro-2H-pyran-2-yl)oxy)-[1,1'-biphenyl]-4-ol (4). 3,4-Dihydro-2H-pyran (1.22 mL, 13.4 mmol) was dropwise added to a solution of 4,4′-dihydroxybiphenyl (5.000 g, 26.9 mmol) and *p*-toluensulphonic acid (0.249 g, 0.2 mmol) in anhydrous THF (30 mL). The reaction mixture was stirred at room temperature for 12 h. Then, the solvent was removed and the residue taken into an aqueous NaOH solution (5 % w/w) (40 mL), extracted with diethyl eter (3×40 mL), the combined organic extracts dried over anhydrous $MgSO_4$ and evaporated to dryness. The residue was purified by column chromatography using hexane/ethyl acetate (4:1 v/v) as eluant. Monoprotected 4,4′-dihydroxybiphenyl (4) was obtained as a white solid (52% yield). FTIR (KBr disk, cm^{-1}): 3343 (O–H). ^{1}H NMR (400 MHz, DMSO-*d6*, δ, ppm): 9.45 (broad s, 1H), 7.50–7.44 (m, 2H), 7.44–7.38 (m, 2H), 7.08–7.02 (m, 2H), 6.84–6.78 (m, 2H), 5.47 (t, *J* = 3.3 Hz, 1H), 3.82–3.72 (m, 1H), 3.60–3.50 (m, 1H), 1.96–1.68 (m, 3H), 1.68–1.46 (m, 3H). ^{13}C NMR (100 MHz, DMSO-*d6,* δ, ppm): 156.61, 155.36, 133.73, 130.71, 127.31, 126.92, 116.78, 115.64, 95.84, 61.56, 29.90, 24.72, 18.67.

Synthesis of 2-((4'-((tetrahydro-2H-pyran-2-yl)oxy)-[1,1'-biphenyl]-4-yl)oxy)ethanol (5). A solution of 2-bromo-1-ethanol (1.949 g, 15.6 mmol) in DMF (20 mL) was dropwise added to a dispersion of compound (4) (3.514 g, 13.0 mmol), 18-crown-6 (0.172 g, 0.65 mmol) and K_2CO_3 (3.593 g, 26.0 mmol) in DMF (20 mL). The reaction mixture was stirred at 120 °C for 24 h. The reaction was quenched with water (100 mL) and extracted with hexane/ethyl acetate (1:1 v/v) (3×100 mL). The combined organic layers were dried over anhydrous $MgSO_4$ and the solvent was evaporated to obtain a viscous liquid that was precipitated in hexane. The residue was purified by recrystallization in ethanol to yield compound (5) as a white solid (2.863 g, 70% yield). FTIR (KBr disk, cm^{-1}): 3389 (O–H). ^{1}H NMR (400 MHz, $CDCl_3$, δ, ppm): 7.51–7.42 (m, 4H), 7.14–7.07 (m, 2H), 7.00–6.94 (m, 2H), 5.45 (t, *J* = 3.1 Hz, 1H), 4.14–4.09 (m, 2H), 4.02–3.89 (m, 3H), 3.67–3.56 (m, 1H), 1.92–1.82 (m, 2H), 1.74–1.58 (m, 4H). NMR ^{13}C (100 MHz, $CDCl_3$, δ, ppm): 157.76, 156.24, 134.22, 134.04, 127.85, 127.66, 116.77, 114.83, 96.46, 69.31, 62.06, 61.54, 30.39, 25.24, 18.80.

Synthesis of (*E*)-2-((4'-((tetrahydro-2H-pyran-2-yl)oxy)-[1,1'-biphenyl]-4-yl)oxy)ethyl 3-(4-methoxyphenyl)acrylate (6). Oxalyl chloride (2.05 mL, 24.0 mmol) was dropwise added to a solution of (*E*)-4-methoxycinnamic acid (2.136 g, 12.0 mmol) and DMF (16 μL, 2.4 mmol) in anhydrous THF (40 mL) refrigerated on ice and under inert atmosphere. The reaction mixture was stirred at room temperature for 4 h. The reaction was then evaporated to dryness and the residue solubilized in anhydrous THF (20 mL). This solution was dropwise added to a solution of compound (5) (2.740 g, 8.6 mmol) and triethylamine (2.785 g, 20.0 mmol) in anhydrous THF (20 mL). The reaction mixture was stirred at room temperature for 18 h and then quenched with an aqueous solution of NH_4Cl (10% w/w) (10 mL). The white precipitate was filtered off and the aqueous phase was extracted with DCM (3×50 mL). The combined organic layers were washed with aqueous NaOH (5% w/w) (2×50 mL), water (50 mL) and a saturated aqueous NaCl solution (50 mL), and dried over anhydrous $MgSO_4$. The solvent was removed and the residue was purified by column chromatography using DCM as eluant. The subsequent recrystallization in ethanol absolute yielded the desired compoun (6) as white crystals (56% yield). FTIR (KBr disk, cm^{-1}): 1708 (C=O), 1203, 1167 (C-O). ^{1}H NMR (400 MHz, $CDCl_3$, δ, ppm): 7.67 (d, J_{trans} = 16.0 Hz, 1H), 7.52 – 7.42 (m, 6H), 7.13 – 7.06 (m, 2H), 7.02 – 6.97 (m, 2H), 6.93 – 6.86 (m, 2H), 6.36 (d, J_{trans} = 16.0 Hz, 1H), 5.45 (t, *J* = 3.2 Hz, 1H), 4.60 – 4.53 (m, 2H), 4.31 – 4.25 (m, 2H), 3.99 – 3.88 (m, 1H), 3.83 (s, 3H), 3.70 – 3.55 (m, 1H), 2.12 – 1.93 (m, 1H), 1.93 – 1.83 (m, 2H), 1.77 – 1.55 (m, 3H). ^{13}C NMR (100 MHz, $CDCl_3$, δ, ppm): 167.21, 161.45, 157.69, 156.19, 145.05, 134.20, 134.00, 129.81, 127.82, 127.66, 127.03, 116.72, 115.05, 114.92, 114.32, 96.40, 66.22, 62.81, 62.04, 55.36, 30.35, 25.21, 18.77.

Synthesis of (*E*)-2-((4'-hydroxy-[1,1'-biphenyl]-4-yl)oxy)ethyl 3-(4-methoxyphenyl)acrylate (7). A solution of the protected compound 6 (2.000 g, 4.2 mmol) and pyridinium p-toluenesulfonate (1.050 g, 4.2 mmol) in THF (150 mL) and ethanol (75 mL) was refluxed for 5 h. The solvent was removed and the residue was solubilized in DCM (75 mL), washed with water (2×75 mL) and brine (75 mL)

and dried over anhydrous $MgSO_4$. Solvent was removed and the solid was thoroughly washed with hexane to yield the corresponding deprotected compound 7 (88% yield). FTIR (KBr disk, cm^{-1}): 3279 (O–H), 1711 (C=O). ^{1}H NMR (400 MHz, $CDCl_3$, δ, ppm): 7.68 (d, J_{trans} = 16.0 Hz, 1H), 7.50–7.44 (m, 4H), 7.44–7.39 (m, 2H), 7.04–6.97 (m, 2H), 6.92–6.86 (m, 4H), 6.36 (d, J_{trans} = 16.0 Hz, 1H), 5.23 (s, 1H), 4.61–4.55 (m, 2H), 4.31–4.26 (m, 2H), 3.84 (s, 3H). ^{13}C NMR (100 MHz, $CDCl_3$, δ, ppm): 167.39, 161.47, 157.63, 154.80, 145.20, 133.93, 133.42, 129.84, 127.94, 127.74, 126.99, 115.61, 114.94, 114.32, 66.21, 62.88, 55.36.

Synthesis of (*E*)-2-((4'-((6-hydroxyhexyl)oxy)-[1,1'-biphenyl]-4-yl)oxy)ethyl 3-(4-methoxyphenyl)acrylate (8). A solution of 6-bromo-1-hexanol (0.623 g, 3.4 mmol) was dropwise added to a dispersion of compound (7) (1.250 g, 3.2 mmol), 18-crown-6 (58.2 mg, 0.2 mmol) and K_2CO_3 (0.885 g, 6.4 mmol) in DMF (10 mL). The reaction mixture was stirred at 120 °C for 24 h. The reaction was diluted with water (40 mL) and extracted with hexane/ethyl acetate (1:1 v/v) (3×40 mL). The combined organic extracts were washed with water (2×100 mL) and a saturated NaCl aqueous solution (100 mL), dried over anhydrous $MgSO_4$ and evaporated to dryness. The residue was purified by column chromatography using hexane/ethyl acetate (4:1 v/v) as eluant to yield the target compound as a white solid (70% yield). FTIR (KBr disk, cm^{-1}): 3348 (O–H), 1720 (C=O). ^{1}H NMR (400 MHz, $CDCl_3$, δ, ppm): 7.67 (d, J_{trans} = 16.0 Hz, 1H), 7.52–7.41 (m, 6H), 7.02–6.97 (m, 2H), 6.97–6.92 (m, 2H), 6.92–6.86 (m, 2H), 6.36 (d, J_{trans} = 16.0 Hz, 1H), 4.60–4.53 (m, 2H), 4.32–4.25 (m, 2H), 4.00 (t, J = 6.5 Hz, 2H), 3.84 (s, 3H), 3.67 (t, J = 6.1 Hz, 1H), 1.86–1.77 (m, 2H), 1.66–1.56 (m, 2H), 1.56–1.41 (m, 4H), 1.32–1.23 (m, 2H). ^{13}C NMR (100 MHz, $CDCl_3$, δ, ppm): 167.19, 161.46, 158.25, 157.64, 145.04, 134.01, 133.17, 129.80, 127.74, 127.70 127.04, 115.07, 114.95, 114.74, 114.33, 67.91, 66.91, 62.91, 62.82, 55.37, 32.69, 29.26, 25.90, 25.54.

Synthesis of (*E*)-2-((4'-((6-azidohexyl)oxy)-[1,1'-biphenyl]-4-yl)oxy)ethyl 3-(4-methoxyphenyl)acrylate (9). A solution of tosyl chloride (0.778 g, 4.1 mmol) in anhydrous DCM (10 mL) was dropwise added to a solution of compound (8) (1.000 g, 2.0 mmol) and triethylamine (0.6 mL, 4.1 mmol) in anhydrous DCM (10 mL) cooled in a ice bath under inert atmosphere. The reaction was kept stirred at room temperature for 18 h. Then, the mixture was washed with diluted HCl (10% w/w) (2×20mL) and saturated NaCl aqueous solution (20 mL), and dried over anhydrous $MgSO_4$. The solvent was removed to obtain the corresponding tosylate intermediate as a white solid (97% yield). FTIR (KBr disk, cm^{-1}): 1697 (C=O), 1407, (SO_2). ^{1}H NMR (400 MHz, $CDCl_3$, δ, ppm): 7.85–7.75 (m, 2H), 7.68 (d, J_{trans} = 16.0 Hz, 1H), 7.55–7.42 (m, 6H), 7.38–7.30 (m, 2H), 7.04–6.95 (m, 2H), 6.94–6.83 (m, 4H), 6.36 (d, J_{trans} = 16.0 Hz, 1H), 4.51–4.45 (m, 2H), 4.33–4.24 (m 2H), 4.05 (t, J = 6.4 Hz, 2H), 3.95 (t, J = 6.4 Hz, 2H), 3.84 (s, 3H), 2.44 (s, 3H), 1.90–1.62 (m, 4H), 1.49 – 1.33 (m, 4H). ^{13}C NMR (100 MHz, $CDCl_3$, δ, ppm): 167.21, 161.46, 158.16, 157.65, 145.05, 144.66, 133.97, 133.23, 129.82, 127.87, 127.74, 127.71, 127.03, 115.05, 114.94, 114.70, 114.32, 70.47, 67.69, 66.23, 62.80, 55.37, 29.04, 28.76, 25.48, 25.16, 21.62. A dispersion of the tosylate intermediate (1.290 g, 2.0 mmol) and sodium azide (0.360 g, 6.0 mmol) in DMF (40 mL) was stirred at 120 °C for 24 h. The reaction mixture was diluted with water (150 mL) and extracted with hexane/ethyl acetate (1:1 v/v) (350 mL). The combined organic layers were washed with water (2×100 mL) and saturated NaCl aqueous solution (100 mL), and dried over anhydrous $MgSO_4$. Volatiles were removed and the solid yellow residue was purified by column chromatography using hexane/ethyl acetate (4:1 v/v) as eluent. Recrystallization in ethanol absolute yielded compound (9) as white crystals (0.823 g, 80% yield). FTIR (KBr disk, cm^{-1}): 2095 (N_3), 1707 (C=O). ^{1}H NMR (400 MHz, $CDCl_3$, δ, ppm): 7.68 (d, J_{trans} = 16.0 Hz, 1H), 7.53–7.39 (m, 6H), 7.04–6.97 (m, 2H), 6.97–6.86 (m, 4H), 6.36 (d, J_{trans} = 16.0 Hz, 1H), 4.60–4.54 (m, 2H), 4.31–4.26 (m, 2H), 4.00 (t, J = 6.4 Hz, 2H), 3.84 (s, 3H), 3.29 (t, J = 6.9 Hz, 2H), 1.88–1.77 (m, 2H), 1.72–1.59 (m, 2H), 1.55–1.41 (m, 4H). ^{13}C NMR (100 MHz, $CDCl_3$, δ, ppm): 167.21, 161.46, 158.21, 157.65, 145.05, 133.98, 133.22, 129.81, 127.75, 127.72, 127.04, 115.06, 114.94, 114.72, 114.32, 67.78, 66.23, 62.81, 55.37, 51.38, 29.15, 28.79, 26.50, 25.69.

Synthesis of (*E*)-2-((4'-((6-aminohexyl)oxy)-[1,1'-biphenyl]-4-yl)oxy)ethyl 3-(4-methoxyphenyl)acrylate (10). A solution of azide (9) (0.523 g, 1.0 mmol) and triphenylphosphine (0.319 g, 1.2 mmol) in anhydrous THF (40 mL) was refluxed for 4 h under inert atmosphere. Reaction mixture was diluted with water (0.2 mL) and stirred at room temperature for 3 h. Volatiles were evaporated and the resulting solid was thorougly washed with hexane/ethyl acetate (7:3 v/v) to yield the target amine (10) as a white solid (97% yield). FTIR (KBr disk, cm^{-1}): 3408, 3354 (N–H), 1701 (C=O). ^{1}H NMR (400 MHz, $CDCl_3$, δ, ppm): 7.67 (d, J_{trans} = 16.0 Hz, 1H), 7.54–7.41 (m, 6H), 7.04–6.97 (m, 2H), 6.96–6.84 (m, 4H), 6.36 (d, J_{trans} = 16.0 Hz, 1H), 4.57 (t, *J* = 4.5 Hz, 2H), 4.28 (t, *J* = 4.5 Hz, 2H), 3.99 (t, *J* = 6.5 Hz, 2H), 3.84 (s, 3H), 2.71 (t, *J* = 6.8 Hz, 2H), 1.88–1.76 (m, 2H), 1.50–1.35 (m, 6H). ^{13}C NMR (100 MHz, $CDCl_3$, δ, ppm): 167.23, 161.51, 157.64, 157.38, 145.05, 134.02, 133.15, 129.82, 127.86, 127.75, 127.04, 115.07, 114.94, 114.74, 114.33, 67.95, 66.23, 62.81, 55.37, 42.14, 33.72, 29.28, 26.67, 25.96.

Synthesis of **N_3-10cCIN/OH.** This intermediate azide was prepared following the procedure described for the synthesis of **N_3-10cCNB/OH** from the cyclic carbonate N_3-bisMPA-carbonate and amine (10). The desired compound was isolated by recrystallizing in ethanol absolute (63% yield). FTIR (KBr disk, cm^{-1}): 3395 (O–H), 2107 (C≡N), 1735 (C=O), 1706 (C=O). ^{1}H NMR (400 MHz, $CDCl_3$, δ, ppm): 7.67 (d, J_{trans} = 16.0 Hz, 1H), 7.57–7.37 (m, 6H), 7.05–6.97 (m, 2H), 6.97–6.83 (m, 4H), 6.36 (d, J_{trans} = 16.0 Hz, 1H), 4.82 (t, *J* = 5.4 Hz, 1H), 4.57 (t, *J* = 5.4 Hz, 2H), 4.36 (d, J_{gem} = 11.6 Hz, 1H), 4.35–4.23 (m, 4H), 4.24 (d, J_{gem} = 11.6 Hz, 1H), 3.99 (t, *J* = 6.4 Hz, 2H), 3.84 (s, 3H), 3.77–3.56 (m, 2H), 3.49 (t, *J* = 5.4 Hz, 3H), 3.28–3.03 (m, 4H), 1.92–1.71 (m, 2H), 1.55–1.44 (m, 4H), 1.45–1.33 (m, 2H), 1.23 (s, 3H). ^{13}C NMR (100 MHz, $CDCl_3$, δ, ppm): 167.20, 161.45, 158.19, 157.63, 156.91, 145.04, 133.97, 133.18, 129.80, 127.73, 127.70, 127.02, 115.15, 114.93, 114.72, 114.32, 72.84, 68.67, 67.80, 66.22, 65.54, 64.80, 64.73, 64.09, 63.61, 63.45, 62.81, 55.36, 49.79, 49.54, 48.90, 41.09, 29.79, 29.16, 26.45, 25.74, 17.46, 17.32, 14.19.

Synthesis of **N_3-7cCIN/eAZO.** The target **N_3-7cCIN/eAZO** was prepared from intermediate **N_3-10cCIN/OH** and 4-(4′-cyanophenylazophenyloxy)octanoic acid as described for **N_3-10cCNB/AZO**. The reaction crude was purified by column chromatography using hexane/ethyl acetate as eluent and **N_3-7cCIN/AZO** was obtained as a red viscous liquid that slowly crystallizes at room temperature (76% yield). FTIR (NaCl window, cm^{-1}): 3337 (N–H), 2225 (C≡N), 2105 (N_3), 1736 (C=O), 1709 (C=O). ^{1}H NMR (400 MHz, $CDCl_3$, δ, ppm): 7.96–7.90 (m, 4H), 7.81–7.74 (m, 2H), 7.67 (d, J_{trans} = 16.0 Hz, 1H), 7.51–7.41 (m, 6H), 7.04–6.96 (m, 4H), 6.96–6.86 (m, 4H), 6.35 (d, J_{trans} = 16.0 Hz, 1H), 4.76 (t, *J* = 5.6 Hz, 1H), 4.56 (t, *J* = 4.0 Hz, 1H), 4.34–4.17 (m, 8H), 4.04 (t, *J* = 6.5 Hz, 2H), 3.97 (t, *J* = 6.4 Hz, 2H), 3.83 (s, 3H), 3.47 (t, *J* = 4.9 Hz, 1H), 3.17 (td, *J* = 6.4, 5.6 Hz, 2H), 2.33 (t, *J* = 7.5 Hz, 2H), 1.89–1.74 (m, 4H), 1.69–1.57 (m, 4H), 1.57–1.44 (m, 6H), 1.43–1.31 (m, 6H), 1.27 (s, 3H). ^{13}C NMR (100 MHz, $CDCl_3$, δ, ppm): 173.21, 172.77, 167.18, 162.67, 161.43, 158.19, 157.62, 155.78, 154.76, 146.66, 145.03, 133.94, 133.16, 133.12, 129.79, 127.71, 127.67, 127.01, 125.44, 123.03, 118.65, 115.03, 114.92, 114.84, 114.70, 114.30, 113.08, 68.34, 67.80, 66.20, 65.79, 65.24, 63.65, 62.79, 55.35, 49.67, 46.71, 41.00, 34.02, 29.82, 29.16, 29.04, 28.96, 26.47, 25.80, 25.75, 24.74, 17.54. HRMS (ESI+) *m*/*z* Exp.: (calc. for $C_{59}H_{68}N_7O_{12}^+$) 1066.4847 (1066.4920). Elem. Anal. Exp. (calc. for $C_{59}H_{67}N_7O_{12}$): C 66.61% (66.46%), H 6.32% (6.33%), N 9,13% (9.20%)

References

1. J. del Barrio, L. Oriol, R. Alcalá, C. Sánchez, *Macromolecules* 2009, **42**, 5752–5760
2. J. Royes, C. Provenzano, P. Pagliusi, R. M. Tejedor, M. Piñol, L. Oriol, *Macromol. Rapid Commun.* 2014, **35**, 1890–1895

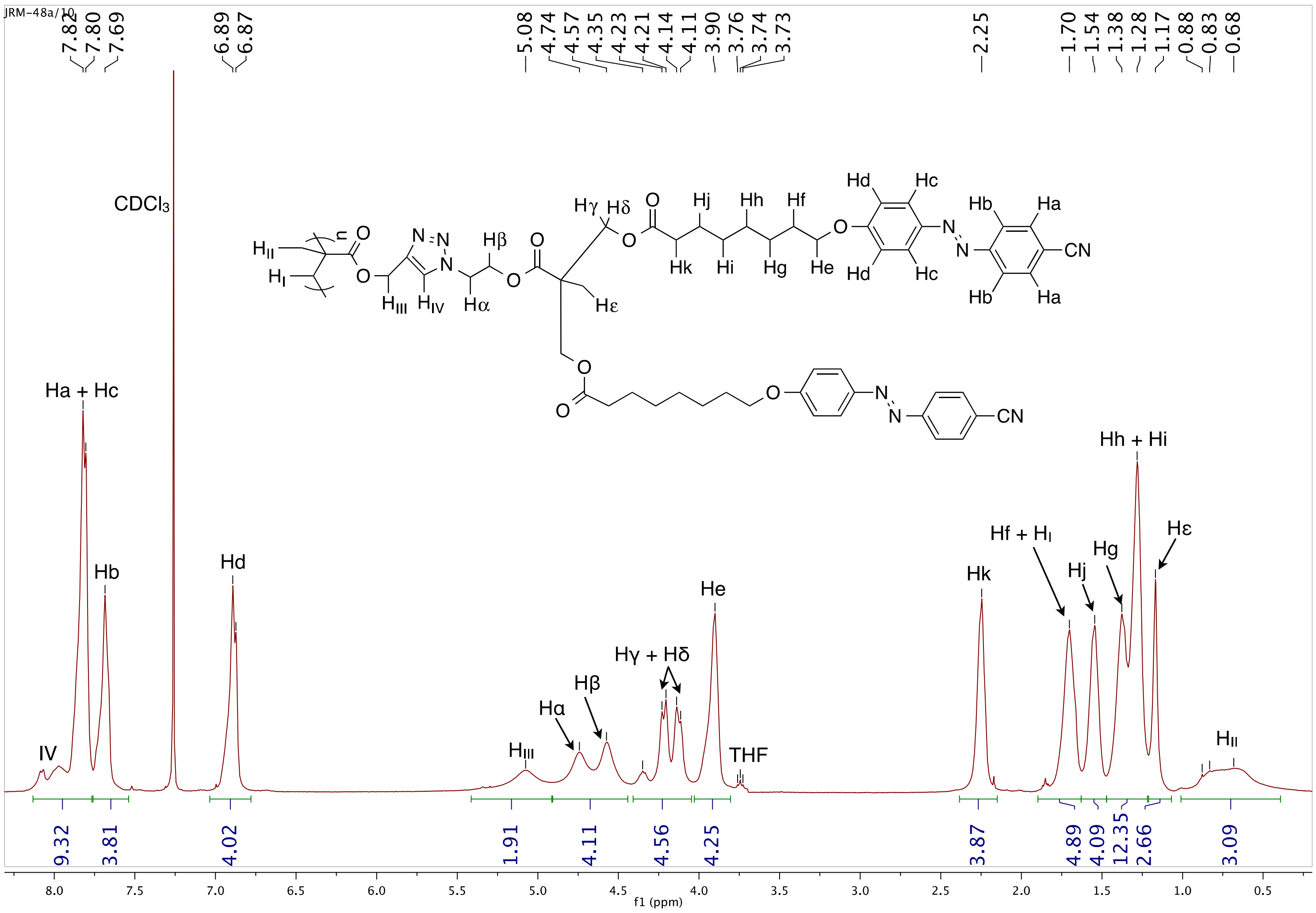

Figure S1. ^{1}H NMR (400 MHz, $CDCl_3$) spectrum of **P_{100}(7eAZO/eAZO)**

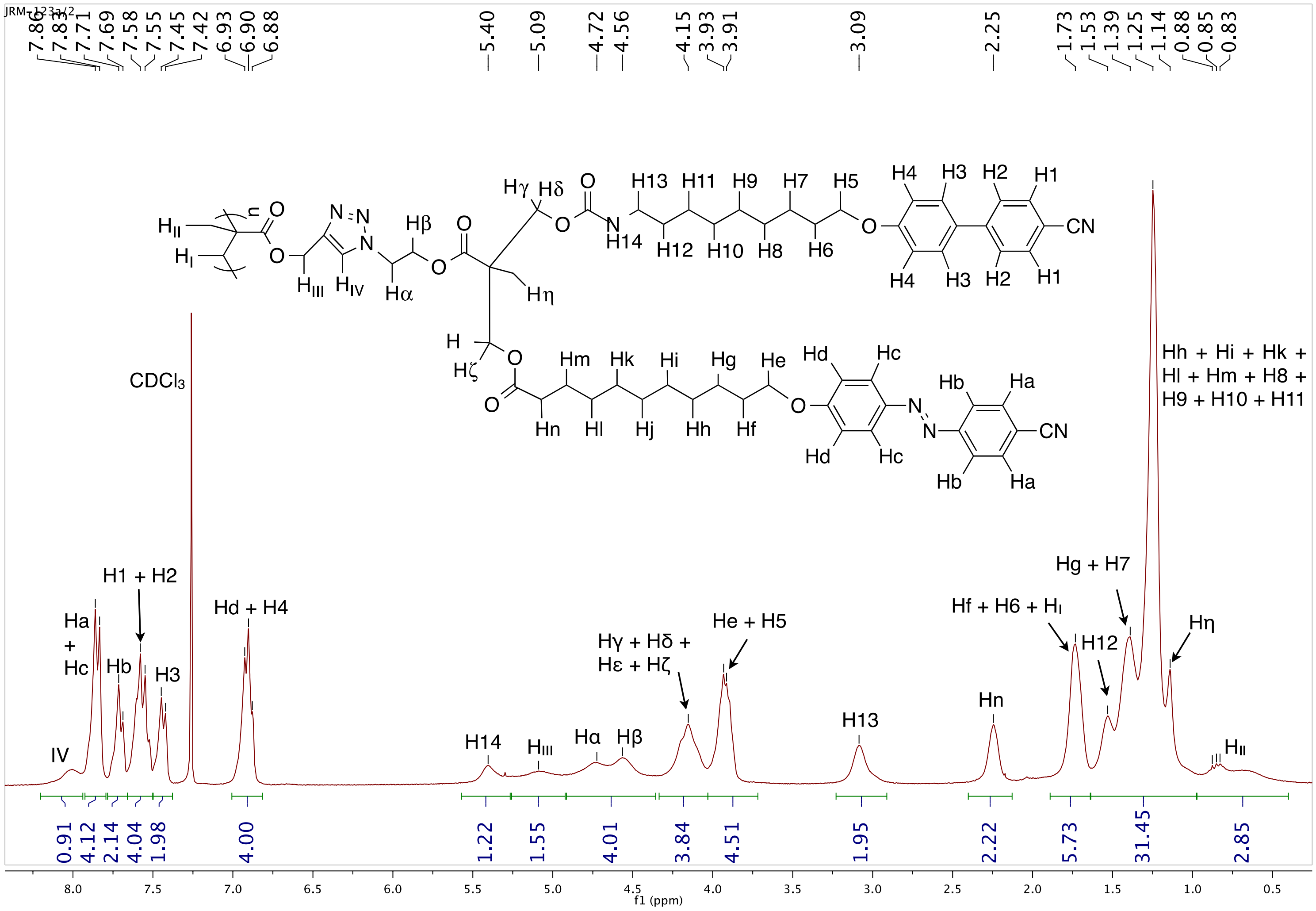

Figure S2. ^{1}H NMR (400 MHz, $CDCl_3$) spectrum of **P_{100}(10cCNB/eAZO)**

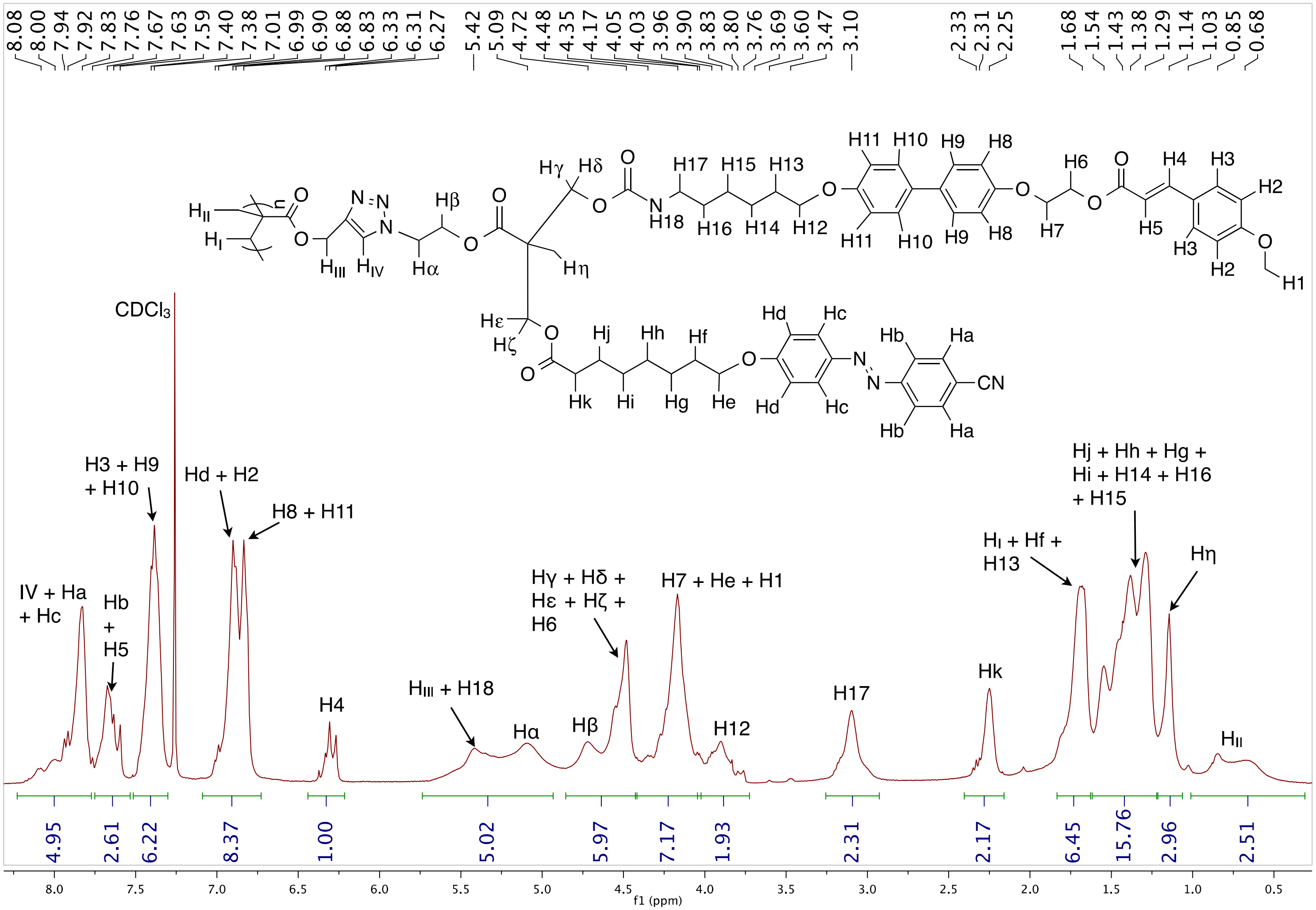

Figure S3. ^{1}H NMR (400 MHz, $CDCl_3$) spectrum of **P_{70}(7cCIN/eAZO)**

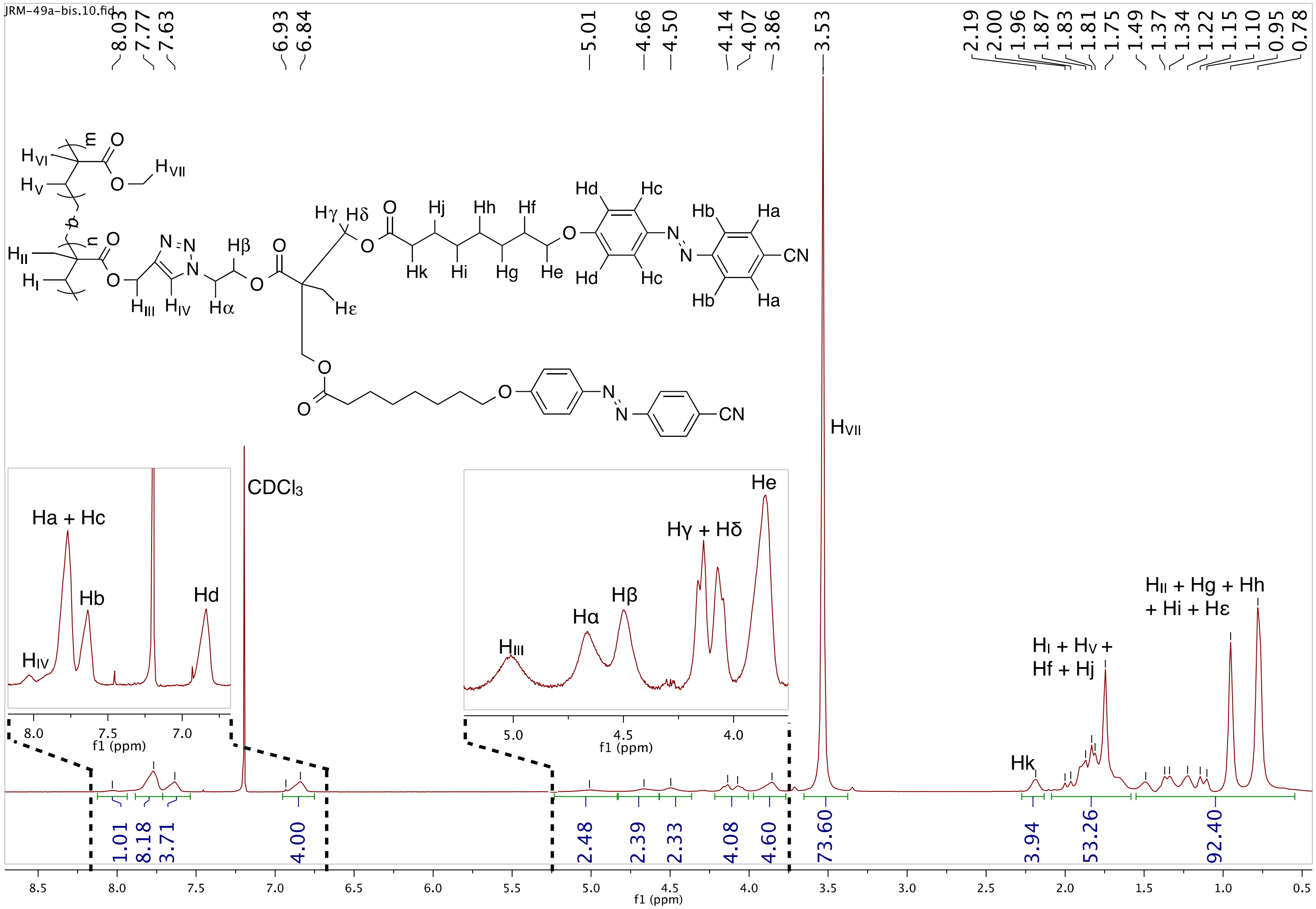

Figure S4. ^{1}H NMR (400 MHz, $CDCl_3$) spectrum of **P_{40}(7eAZO/eAZO)-*b*-$PMMA_{800}$**

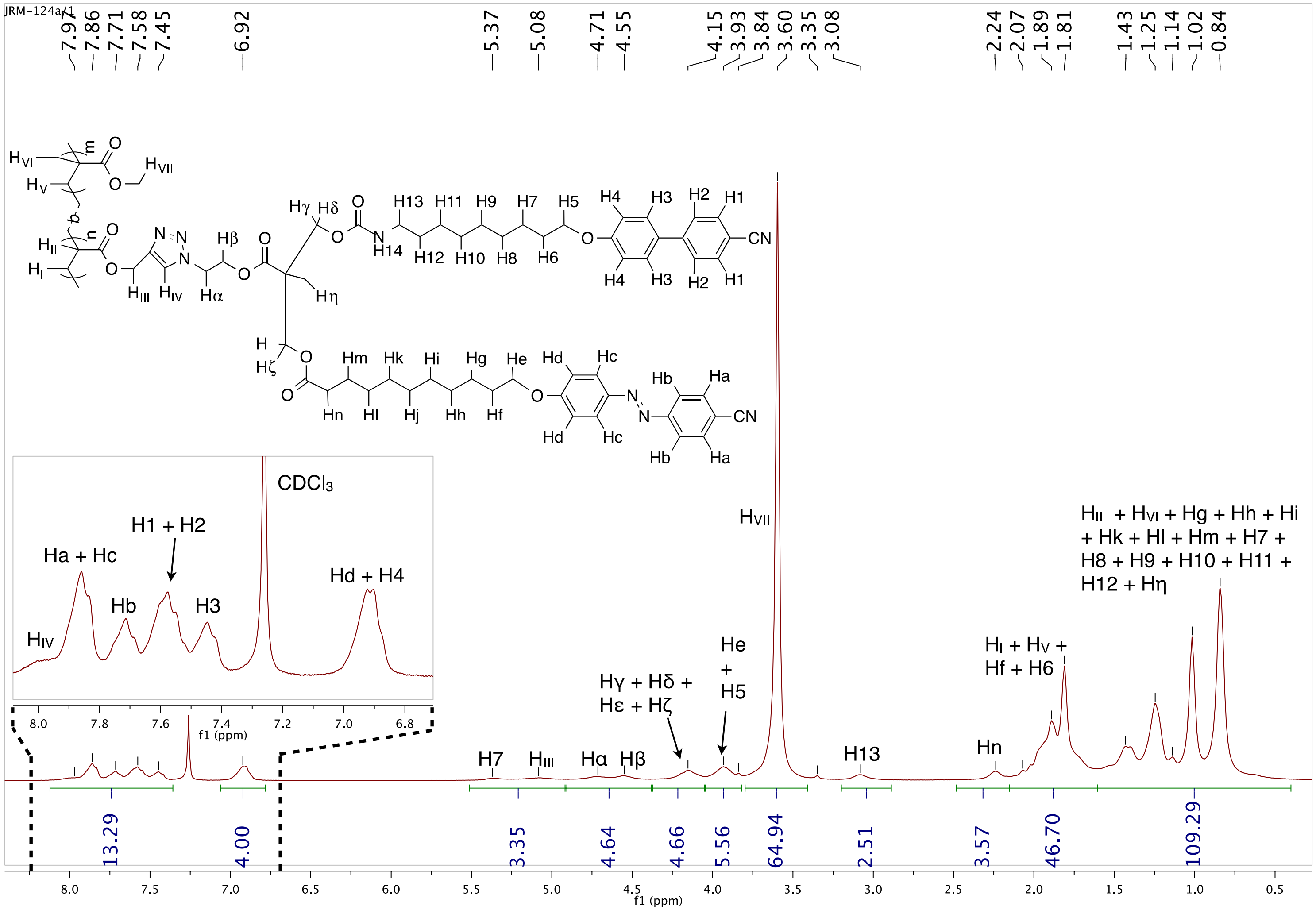

Figure S5. ^{1}H NMR (400 MHz, $CDCl_3$) spectrum of **P_{40}(9cCNB/eAZO)-*b*-$PMMA_{800}$**

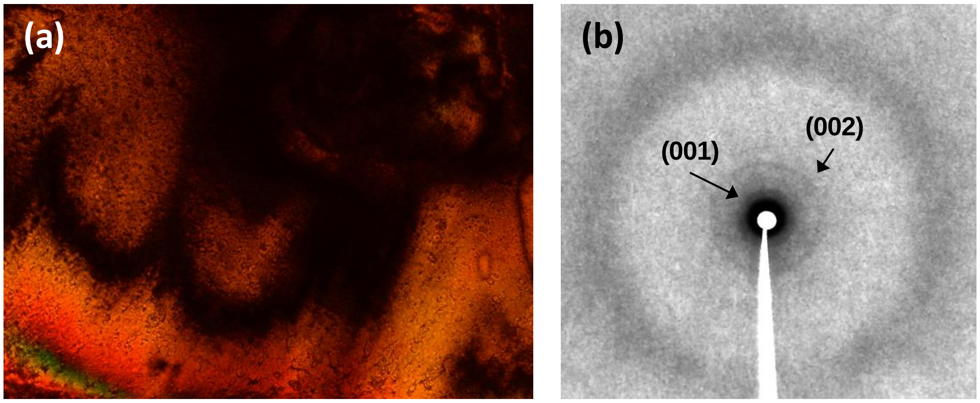

Figure S6. (a) POM photograph taken at 125 °C on cooling from isotropic liquid and (b) XRD difractogram registered at room temperature after annealing at 96 °C of **P$_{40}$(7eAZO/eAZO)-*b*-PMMA$_{800}$**

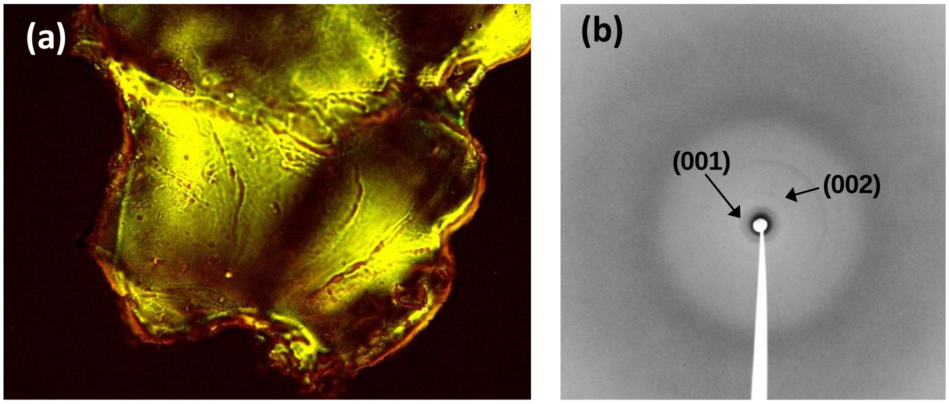

Figure S7. (a) POM photograph taken at 105 °C on cooling from isotropic liquid and (b) XRD difractogram registered at room temperature after annealing at 80 °C of **P$_{40}$(10cCNB/eAZO)-*b*-PMMA$_{800}$**

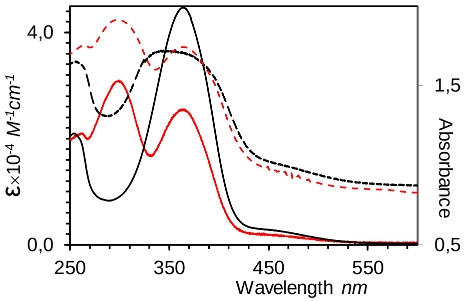

Figure S8. UV-vis spectra in THF solution (solid lines) and thin film (dotted lines) of **P$_{40}$(7eAZO/eAZO)-*b*-PMMA$_{800}$** (black lines) and **P$_{40}$(10cCNB/eAZO)-*b*-PMMA$_{800}$**

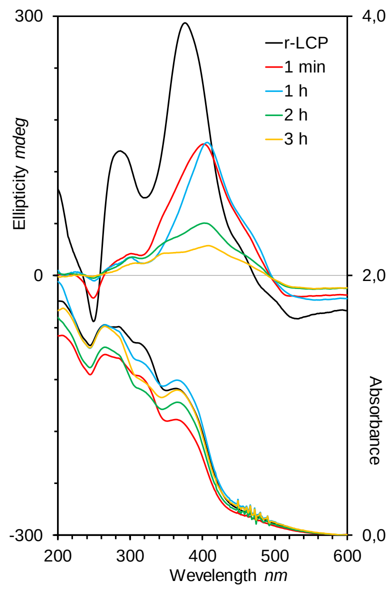

Figure S9. UV-vis and ECD spectra of **P$_{70}$(7cCIN/eAZO)** film after r-CPL irradiation (black) and r-CPL irradiated film exposed at UV light for 1 min, 1 h, 2 h and 3 h
